# Leveraging polygenic risk scores to infer causal directions in genotype-by-environment interactions between complex traits

**DOI:** 10.1007/s00439-025-02799-x

**Published:** 2026-02-07

**Authors:** Zinabu Fentaw, Buu Truong, Dovini Jayasinghe, Chris Della Vedova, Gibran Hemani, Beben Benyamin, Elina Hyppönen, S. Hong Lee

**Affiliations:** 1 Australian Centre for Precision Health, University of South Australia, Adelaide, SA Australia; 2 UniSA Allied Health and Human Performance, University of South Australia, Adelaide, SA Australia; 3 South Australian Health and Medical Research Institute (SAHMRI), University of South Australia, Adelaide, SA Australia; 4 Department of Epidemiology and Biostatistics, School of Public Health, Wollo University, Dessie, Ethiopia; 5 Program in Medical and Population Genetics and the Cardiovascular Disease Initiative, Broad Institute of MIT and Harvard, 415 Main St, Cambridge, MA 02142 USA; 6 Center for Genomic Medicine and Cardiovascular Research Center, Massachusetts General Hospital, 185 Cambridge Street, Boston, MA 02114 USA; 7 UniSA Clinical & Health Sciences, University of South Australia, Adelaide, SA 5001 Australia; 8 Medical Research Council Integrative Epidemiology Unit, University of Bristol, Bristol, UK; 9 Population Health Sciences, Bristol Medical School, University of Bristol, Bristol, UK

## Abstract

Most existing genotype-by-environment interaction (G×E) methods assume a known causal direction as an assumption that often does not hold and can lead to biased estimates and spurious findings. To address this, we introduce the Genetic Causality Inference Model (GCIM), a novel approach designed to infer causal directions in G×E studies. GCIM integrates polygenic risk scores (PRS) for both the exposure and the outcome to strengthen causal inference and reduce spurious interaction signals. We evaluated GCIM using simulated data across varying genetic and residual correlation settings and compared its performance to existing PRS-by-environment (PRS×E) models under both null and alternative G×E scenarios. GCIM was also applied to real-world UK Biobank data in both causal directions. GCIM consistently outperformed existing methods by accurately identifying the absence of G×E variance and avoiding false positives, even in the presence of strong phenotypic heteroscedasticity due to residual heterogeneity. Other methods often generated spurious associations, especially under reverse causality. Applying GCIM to UK Biobank data, we investigated 11 circulating biomarkers (including liver enzymes, lipids, and inflammatory markers) and three anthropometric traits (BMI, body fat, and waist-to-hip ratio [WHR]). GCIM identified that bilirubin modulates genetic effects on BMI and WHR, while body fat modulates genetic effects on C-reactive protein, with associations remaining significant after multiple testing corrections. Overall, GCIM provides a more reliable framework for GxE analysis, particularly under challenging conditions such as residual heterogeneity and uncertain causal direction. However, further development is needed to improve its statistical power.

**Supplementary Information:**

The online version contains supplementary material available at 10.1007/s00439-025-02799-x.

## Introductions

Complex diseases, such as diabetes, cardiovascular disorders, and psychiatric conditions, arise from the combined influence of multiple genetic and environmental factors. Understanding the intricate mechanisms underlying these diseases is critical for improving prevention, diagnosis, and treatment strategies. Traditionally, research has focused on identifying genetic variants or environmental exposures individually, but it has become increasingly clear that their interaction plays a crucial role in shaping disease risk and phenotypic variability. This interplay, known as genotype-by-environment interaction (G×E), reflects how genetic effects can be modified by environmental factors, and how environmental effects can also be influenced by genetic variation, offering deeper insights into disease aetiology (Favé et al. 2018; Gage et al. 2016).

A wide array of statistical methods has been developed to disentangle the contributions of genetic and environmental factors, as well as their interaction (Jayasinghe et al. 2024; Shin and Lee 2021). Existing models typically estimate the main effects of genetic variants and environmental exposures separately (Bogdan et al. 2018; Burton et al. 2007) or incorporate an interaction term to model their joint effect (Miao et al. 2024; Moore et al. 2019; Shin and Lee 2021). Genome-wide environment interaction studies (GWEIS) are also used to identify genetic variants whose effects are modified by environmental factors (Murcray et al. 2009). Advances in genome-wide technologies and the increasing availability of large biobanks have further enabled construction of polygenic risk score (PRS) and the application in G×E analyses, often referred to as PRS×E models (Jayasinghe et al. 2024; Plomin et al. 2022; Tang et al. 2022). These approaches aggregate the effects of many genetic variants into a single risk score, thereby increasing power and interpretability. Initially, PRS×E models typically used PRS derived from GWAS of the outcome trait (Byrne et al. 2023; Durvasula and Price 2025; Miao et al. 2025; Plomin et al. 2022). However, these approaches were limited by reduced power and inflated false positive rates due to model misspecification. To address this, later methods used PRS based on interaction effects estimated in GWEIS (Tang et al. 2022), but these too suffer from inflated Type I error rates. The recently proposed GxEprs method (Jayasinghe et al. 2024) overcomes these limitations by jointly modelling the main and interaction effects of PRS based on interaction effects estimated in GWEIS, thereby mitigating problems caused by model misspecification.

Despite the methodological progress, critical limitations remain in the current landscape of G×E research. One particularly underexplored challenge is the determination of causal direction of GxE (Jayasinghe et al. 2024; Shin and Lee 2021). While most statistical methods assume a fixed causal direction, typically treating environmental variables as exposures and phenotypes as outcomes, this assumption is often untested. This issue is especially problematic when environmental variables, such as smoking, alcohol consumption, or education themselves have genetic components. For example, in the context of smoking and depression, most analyses treat smoking status as the environmental exposure modifying genetic effects on depression. However, the reverse direction is equally plausible: depression status could modulate genetic susceptibility to smoking initiation or nicotine dependence. Such bidirectional possibilities illustrate how assuming a fixed causal direction may overlook the complex feedback between environment and phenotype.

Some studies have attempted to address this by switching the roles of exposure and outcome (Aschard et al. 2018; Cheng et al. 2021), not to infer causal direction per se, but to mitigate issues caused by measurement error or confounding. However, such approaches do not resolve the fundamental question of causality and can lead to biased or spurious findings if the true direction is misidentified. Currently, no established methods reliably infer causal direction in G×E analyses. The inability to distinguish whether exposure variables temporally modify genetic effects on outcomes or vice versa remains a significant challenge (Thomas 1988; 2010). Consequently, this limitation can result in invalid conclusions and hinder the effective application of findings, ultimately undermining the reliability and utility of these studies in understanding the complex relation between genetic and environmental factors. Addressing this challenge requires the development of robust methods capable of reliably inferring causal direction and reducing biased conclusion from spurious associations.

The need for such methodology is increasingly urgent as biobank-scale datasets enable testing of hundreds of potential G×E. Without proper causal direction inference, the field risks accumulating false positive findings that may misdirect therapeutic development and personalized medicine efforts. In response to these challenges, we propose a genetic causality inference model (GCIM), a novel statistical method that advances beyond existing PRSxE approaches in three key ways: (1) it systematically tests both proposed and reverse causal directions, (2) it uses polygenic risk scores of the exposure trait instead of observed phenotypes to eliminate non-zero covariances between the exposure and the G×E component of the outcome during reverse-direction testing, which would otherwise introduce bias when a true causal effect exists in the proposed direction, and (3) it provides a formal framework for distinguishing true G×E signals from artifacts of causal misspecification. Unlike traditional methods that assume causal direction a priori, GCIM explicitly evaluates the evidence for each direction, providing researchers with data-driven guidance for causal inference.

## Methods

### Statistical models

We employ PRSxE models (Jayasinghe et al. 2024) to determine causal directions. To apply PRSxE models, we split the total sample (n, representing the total number of individuals) into two independent, non-overlapping datasets: 80% was designated as the discovery dataset (n_d_), while the remaining 20% served as the target dataset (n_t_). Both datasets contain outcome, exposure variables, and genotypic data. The discovery dataset was used to generate genome-wide-by-environment interaction study (GWEIS) summary statistics, which were subsequently applied to compute PRS for both main genetic effects ($\mathbf{\hat{g}}_{{\mathbf{y}}}$) and non-additive genetic effects ( $\mathbf{\hat{g}}_{{{\mathbf{y}}_{{{\mathbf{g \times e}}}} }}$). The model can be written as:

1 $${\mathbf{y}}_{{\mathbf{d}}} {\boldsymbol=} {\text{ }}{\mathbf{X}}{\boldsymbol{\beta }}_{{{\mathbf{add}}}} {\boldsymbol{ + }}{\text{ }}{\mathbf{c}}_{{\mathbf{d}}}{\beta }_{{{{c}}}}{\boldsymbol{ + }}{\text{ }}({\mathbf{X}}{\boldsymbol{ \odot }}{\mathbf{c}}_{{\mathbf{d}}} ){\mathrm{ }}{\boldsymbol{\beta }}_{{{\mathbf{g \times e}}}} {\boldsymbol+} {\mathrm{ }}\boldsymbol{\varepsilon }_{{{\mathbf{y}}}} $$

where **y**_**d**_ and **c**_**d**_ represent the n_d_ × 1 vectors of outcome and exposure, in the discovery dataset, respectively. **X** is the n_d_ × m genotype matrix, where *m* is the number of single nucleotide polymorphisms (SNPs). **β**_**add**_ and **β**_**gxe**_ represent the additive and non-additive genetic effects of the m SNPs, ${\beta}_{{{{c}}}}$ is the estimated effect of the exposure on $\:{\mathbf{y}}_{\mathbf{d}}$, and $\boldsymbol{\varepsilon }_{{{\mathbf{y}}}}$ is the n_d_ × 1 vector of residual effects. The Hadamard product (⊙) signifies element-wise multiplication of vectors.    

Using the GWEIS results, the two sets of PRS for the outcome in any given sample was computed as:

2 $${\hat{g}}_{{y_{i} }} \: = \:\sum\nolimits_{{j = 1}}^{m} {W_{{ij}} \hat{\beta }_{{add_{j} }} } $$

and

3 $${\hat{g}}_{{y_{{g \times e_{i} }} }} \: = \:\sum\nolimits_{{j = 1}}^{m} {W_{{ij}} \hat{\beta }_{{g \times e_{j} }} } $$

where ${\hat{g}}_{{y_{i} }}$ and ${\hat{g}}_{{y_{{g \times e_{i} }} }}$ are the estimated PRSs constructed using additive genetic effects and the GxE effects on the outcome for the i^th^ individual in the target dataset, respectively. $\:{W}_{ij}$ represents the genotype of the i^th^ individual at j^th^ SNP in n_t_ × m of genotype matrix in the target dataset. $\hat{\beta }_{{add_{j} }}$ and $\hat{\beta }_{{g\times e_{j} }}$ are the estimated additive and interaction genetic effects of the j^th^ SNP, as obtained from the GWEIS (Eq. 1).            

Additionally, we use the discovery dataset to generate genome-wide association studies (GWAS) summary statistics for the exposure, then compute the PRS for the exposure (**ĝ**_**c**_). The model can be written as, assuming no interaction terms in the modelling of the exposure phenotypes:

4 $${\mathbf{c}}_{{\mathbf{d}}} {\boldsymbol{ = }}~{\mathbf{X}}\boldsymbol{\beta }_{{{\mathbf{add}}_{{\mathbf{c}}} ~}} {\boldsymbol{ + }}\boldsymbol{~\varepsilon }_{{\mathbf{c}}}$$

where $\boldsymbol{\beta }_{{{\mathbf{add}}_{{\mathbf{c}}} \:}}$represents the estimated genetic effects of the m SNP for the exposure. X includes the genotypic information, as defined earlier. $~\boldsymbol{\varepsilon }_{{\mathbf{c}}}$ is the n_d_ × 1 vector of residual effects.

The PRS of the main genetic effects on the exposure for an individual was computed as:

5 $$ \hat{g}_{{c_{i} }} = ~\sum\nolimits_{{j = 1}}^{m} {W_{{ij}} \hat{\beta }_{{add_{{c_{j} }} ~}} } $$

where $\hat{g}_{{c_{i} }}$ is the estimated PRS of the genetic effect of exposure for the i^th^ individual in the target dataset. $\:{W}_{ij}$ includes the genotypic information, as defined earlier. ${\hat{\beta }_{{add_{{c_{j} }} ~}} }$represents the estimated effect of the j^th^ SNP on the exposure, obtained from the GWAS using the discovery dataset (Eq. 4).

Subsequently, using the target dataset, the significance of PRSxE effects can be tested the method by Plomin et al. (Plomin et al. 2022). The model can be written as:

Model 1 $${\mathbf{y}}_{{\mathbf{t}}} {\boldsymbol=} \beta _{{\mathrm{1}}} \mathbf{\hat{g}}_{{\mathbf{y}}} {\boldsymbol+} \beta _{{\mathrm{2}}} {\mathbf{c}}_{{\mathbf{t}}} {\boldsymbol+} \beta _{{\mathrm{3}}} \left( {\mathbf{\hat{g}}_{{\mathbf{y}}} {\boldsymbol{ \odot }} {\mathbf{c}}_{{\mathbf{t}}} } \right) {\boldsymbol+} {\text{ }}{\mathbf{e}}_{{\mathbf{y}}}$$

where $\:{\mathbf{y}}_{\mathbf{t}}$ and $\:{\mathbf{c}}_{\mathbf{t}}$ are the outcome and exposure variables in the target dataset, respectively. **ĝ**_**y**_ represents the n_t_ x 1 estimated PRSs for the main genetic effects (Eq. 2). However, it is noted that **ĝ**_**y**_ in Model 1 is derived from the GWAS, not the GWEIS, for the outcome in the discovery dataset. *β*_*1*_, *β*_*2*_, and *β*_*3*_ represent the regression coefficients of an additive genetic effects, the observational effect of the exposure, and the PRSxE effects, respectively. **e**_**y**_ represents the residual effects on **y**_**t**_.

Tang et al. (Tang et al. 2022) developed a new model using the PRSxE in the following ways:

Model 2 $${\mathbf{y}}_{{\mathbf{t}}} = \beta _{1} \mathbf{\hat{g}}_{{\mathbf{y}}} + \beta _{2} {\mathbf{c}}_{{\mathbf{t}}} + \beta _{{\mathrm{3}}} ({\mathbf{\hat{g}}}_{{\mathbf{y}_{{\mathbf{g \times e}}} }} {\boldsymbol{ \odot }} {\mathbf{c}}_{{\mathbf{t}}} ) + {\mathbf{e}}_{{\mathbf{y}}}$$

where **ĝ**_**y**_ represents the n_t_ x 1 estimated PRSs for the main genetic effects (Eq. 2). $\mathbf{\hat{g}}_{{{\mathbf{y}}_{{{\mathbf{g \times e}}}} }}$represents the estimated PRS of the non-additive effects using Eq. 3 based on the GWEIS using the discovery dataset.

In addition to Models 1 and 2, a more comprehensive approach for modelling PRS×E has been introduced (Jayasinghe et al. 2024) to mitigate false positives and improve statistical power. This model is specified as follows:

Model 3 $${\mathbf{y}}_{{\mathbf{t}}} {\boldsymbol=} \beta _{{\mathrm{1}}} {\mathbf{\hat{g}}}_{{\mathbf{y}}} + \beta _{{\mathrm{2}}} {\mathbf{c}}_{{\mathbf{t}}} + \beta _{{\mathrm{3}}} ~{\mathbf{\hat{g}}}_{{\mathbf{y}_{{\user2{g \times e}}} }} + \beta _{{\mathrm{4}}} ({\mathbf{\hat{g}}}_{{\mathbf{y}_{{\user2{g \times e}}} }} {\boldsymbol{ \odot }} {\mathbf{c}}_{{\mathbf{t}}} ) + {\mathbf{e}}_{{\mathbf{y}}}$$

where all terms remain the same as in Model 2, with the exception of the inclusion of the main effects of the PRS for non-additive effects ($\mathbf{\hat{g}}_{{{\mathbf{y}}_{{{\mathbf{g \times e}}}} }}$) on **y**_**t**_. *β*_*3*_ and *β*_*4*_ represent the regression coefficients of the main effects of the estimated PRS for non-additive effects ($\mathbf{\hat{g}}_{{{\mathbf{y}}_{{{\mathbf{g \times e}}}} }}$) and the PRSxE effects, respectively.    

Finally, Model 4, our proposed model (GCIM), incorporates the estimated PRS of the exposure (Eq. 5), instead of the observed exposure phenotypes. We refer to this model as the GCIM. This approach helps mitigate spurious interaction signals, reducing the risk of false causal inference when testing the causal directions (**y** as the outcome and **c** as the exposure vs. **y** as the exposure and **c** as the outcome) (see Supplementary Note). The GCIM for one direction (**y** as the outcome and **c** as the exposure) can be written as:

Model 4 $${\mathbf{y}}_{{\mathbf{t}}} {\boldsymbol{ = }}\beta _{1} {\mathbf{\hat{g}}}_{{\mathbf{y}}} + \beta _{2} {\mathbf{c}}_{{\mathbf{t}}} + \beta _{3} {\text{ }}{\mathbf{\hat{g}}}_{{{\mathbf{y}}_{{{\mathbf{g \times e}}}} }} {\mathbf{~}} + \beta _{4} \left( {~{\mathbf{\hat{g}}}_{{{\mathbf{y}}_{{{\mathbf{g \times e}}}} }}{\boldsymbol{ \odot }} {\mathbf{\hat{g}}}_{{\mathbf{c}}} } \right){\boldsymbol{ + e}}_{{\mathbf{y}}}$$

where, **ĝ**_**c**_ represents the n_t_ × 1 vector of the estimated PRSs for the main genetic effects on the exposure (Eq. 5) based on the GWAS using the discovery dataset. Models 1–4 can be evaluated under reverse causal directions by switching the roles of exposure and outcome while maintaining consistency in data preparation with the original model structure. We provide an example for Model 4 in the following, but the same approach applies to Models 1–3 (not explicitly shown). For instance, in Model 4 (GCIM) under the reverse causal framework, where **c** is treated as the outcome and **y** as the exposure, the model can be expressed as:

$${\mathbf{c}}_{{\mathbf{t}}} {\boldsymbol{ = }}\beta _{1} {\mathbf{\hat{g}}}_{{\mathbf{c}}} + \beta _{2} {\mathbf{y}}_{{\mathbf{t}}} + \beta _{3} {\text{ }}{\mathbf{\hat{g}}}_{{{\mathbf{c}}_{{{\mathbf{g \times e}}}} }} {\mathbf{~}} + \beta _{4} \left( {~{\mathbf{\hat{g}}}_{{{\mathbf{c}}_{{{\mathbf{g \times e}}}} }}{\boldsymbol{ \odot }} {\mathbf{\hat{g}}}_{{\mathbf{y}}} } \right){\boldsymbol{ + e}}_{{\mathbf{c}}}$$

where **ĝ**_**y**_ represents the estimated PRS for **y**, constructed based on additive SNP effects estimated in the GWAS for **y**, using the discovery dataset (analogous to Eqs. 4 and 5). In the target dataset, $\:{\mathbf{c}}_{\mathbf{t}}$ and $\:{\mathbf{y}}_{\mathbf{t}}$ are modelled as the outcome and exposure variables, respectively. **ĝ**_**c**_ and $\mathbf{\hat{g}}_{{{\mathbf{c}}_{{{\mathbf{g \times e}}}} }}$ represent the PRS of additive and non-additive genetic effects on c, estimated from the GWEIS using the discovery dataset (analogous to Eqs. 1 and 2). The coefficients *β1*,* β2*,* β3*, and *β4* correspond to the additive genetic effect, the observational effect of the exposure, the main effect of the estimated PRS for non-additive interactions, and PRS×E, respectively, in this reverse causation model for Model 4. **e**_**c**_ represents the residual effects on **c**_**t**_.

Among the four models evaluated, Model 4 represents our proposed model, GCIM. GCIM extends existing G×E frameworks by incorporating exposure PRS to infer the causal direction between exposure and outcome. A detailed description of the GCIM framework and its underlying assumptions is provided in the Supplementary file (see Supplementary Note).

### Data source and quality control for genotype data

We compared Models 1–4 using simulated phenotypic data generated from real genotypic data derived from the UK Biobank (https://www.ukbiobank.ac.uk/) (Sudlow et al. 2015). Additionally, we evaluated the proposed method (Model 4, GCIM) using genotypic and phenotypic data from the UK Biobank to assess its performance in a real-world setting. UK Biobank dataset comprises around 92,693,895 SNPs and numerous phenotype variables from approximately 500,000 UK residents enrolled between 2006 and 2010 (Sudlow et al. 2015). The participants were aged between 40 and 69 years old (Ollier et al. 2005). It offers an extensive range of data on each participant, encompassing diverse phenotypic and health-related details such as biological measurements, lifestyle factors, blood and urine biomarkers (Bycroft et al. 2018).

Genotyping was performed using the UK Biobank Axiom Array, with imputation conducted using reference panels from the Haplotype Reference Consortium, UK10K, and 1000 Genomes Project (Auton et al. 2015; Loh et al. 2016; Walter et al. 2015). To minimize genetic heterogeneity, analyses were restricted to the largest ancestry group, the White British population (*n* = 408,183). Rigorous quality control (QC) procedures were applied at both the SNP level and individual level to ensure high-quality genotype data.

At the SNP level, variants with low imputation quality (INFO score < 0.6) (Roshyara et al. 2014), multi-character allele codes, minor allele frequency (MAF) < 0.01, significant deviation from Hardy-Weinberg equilibrium (*p* < 10^− 7^), low call rate (< 95%), or duplicate IDs were excluded. At the individual level, exclusions were made for non-White-British ancestry, high missingness (> 5%), sex mismatches between submitted and inferred sex, poor genotype quality, sex chromosome aneuploidy, and close relatives with relatedness > 0.05 based on the Genomic Relationship Matrix. After applying these QC filters, a total of 7,701,772 SNPs and 288,792 individuals remained for further analyses.

For this study, we specifically utilized HapMap3 SNPs, as they are widely recognized for their robustness and reliability in estimating heritability and genetic correlation (Bulik-Sullivan et al. 2015; Tropf et al. 2017), thereby improving the accuracy of genetic prediction. After these QC steps, the final dataset used in the analyses comprised 1,118,829 SNPs and 288,792 individuals.

### Data simulation for the outcome and exposure

The outcome and exposure variables were simulated using 50,000 individuals and 200,000 SNPs, randomly sampled from the quality-controlled UK Biobank dataset. Among these 200,000 SNPs, 10,000 SNPs were randomly selected and assigned causal effects for **y** and **c** according to the covariance structures specific to each simulation scenario (detailed below). Phenotypic data were simulated with **y** as the outcome and **c** as the exposure variable across all simulation scenarios, and analyses were conducted in both causal directions.

We generated phenotypic data for **y** and **c** under several distinct scenarios, varying the presence of G×E and residual-by-environment interactions (i.e. phenotypic or residual heteroscedasticity) while considering for different levels of genetic and residual correlations. Note that residual heteroscedasticity, as well as genetic and residual correlations often occur in real data and can cause spurious GxE signals.

#### Simulating the null model for GxE

In this scenario, the outcome was simulated based on the Eq. 1 without the main effects of exposure ($\:{\beta\:}_{c}=0$) and GxE (var(**β**_**gxe**_) =0). The exposure variable was generated using Eq. 4. The SNP effects, **β**_**add**_, **β**_**gxe**_ and $\boldsymbol{\beta }_{{{\mathbf{add}}_{{\mathbf{c}}} \:}}$for the 10,000 causal SNPs were drawn from a multivariate normal distribution with a mean of zero and the following variance-covariance structure:

$$\begin{gathered} \left[ {\begin{array}{*{20}c} {\begin{array}{*{20}c} {var\left( {\boldsymbol{\beta }_{{{\mathbf{add}}}} } \right)} & {cov\left( {\boldsymbol{\beta }_{{{\mathbf{add}}}} \boldsymbol{\beta }_{{{\mathbf{g \times e}}}} } \right)} & {cov\left( {\boldsymbol{\beta }_{{{\mathbf{add}}}} ,\boldsymbol{\beta }_{{{\mathbf{add}}_{{\mathbf{c}}} ~}} } \right)} \\ {cov\left( {\boldsymbol{\beta }_{{{\mathbf{add}}}} ,\boldsymbol{\beta }_{{{\mathbf{g \times e}}}} } \right)~} & {var\left( {\boldsymbol{\beta }_{{{\mathbf{g \times e}}}} } \right)} & {cov\left( {\boldsymbol{\beta }_{{{\mathbf{add}}_{{\mathbf{c}}} ~}} ,\boldsymbol{\beta }_{{{\mathbf{g \times e}}}} } \right)} \\ {cov\left( {\boldsymbol{\beta }_{{{\mathbf{add}}}} ,\boldsymbol{\beta }_{{{\mathbf{add}}_{{\mathbf{c}}} ~}} } \right)} & {cov\left( {\boldsymbol{\beta }_{{{\mathbf{add}}_{{\mathbf{c}}} ~}} ,\boldsymbol{\beta }_{{{\mathbf{g \times e}}}} } \right)} & {var\left( {\boldsymbol{\beta }_{{{\mathbf{add}}_{{\mathbf{c}}} ~}} } \right)} \\ \end{array} } \\ \end{array} } \right] \hfill \\ = \left[ {\begin{array}{*{20}c} {\begin{array}{*{20}c} {0.4} & 0 & {0,~0.179,~0.358} \\ 0 & 0 & 0 \\ {0,~0.179,~0.358} & 0 & {0.5} \\ \end{array} } \\ \end{array} } \right] \hfill \\ \end{gathered}$$

where ${\boldsymbol{\beta }_{{{\mathbf{add}}}} }$, ${\boldsymbol{\beta }_{{\mathbf{g \times e}}} }$, and ${\boldsymbol{\beta }_{{{\mathbf{add}}_{{\mathbf{c}}} ~}} }$ are as defined in Eqs. 1 and 4 above.    

Comma-separated values indicate that the corresponding matrix element was systematically varied across simulation scenarios, with each value representing a distinct level of genetic or residual covariance. Each value was substituted independently while preserving the overall matrix structure and dimensionality. This approach is applied consistently across all simulation scenarios for all matrices presented therein.

Based on these SNP effects and the UK Biobank genotypic data, individual-level genetic effects were computed for the outcome ($\:{\mathbf{g}}_{\mathbf{y}}$) and exposure ($\:{\mathbf{g}}_{\boldsymbol{c}}$) (Lee and van der Werf 2016), using Eq. 3 and Eq. 5. The resulting variance-covariance matrix for the individual genetic effects followed the same structure as the SNP effects, as expected:

$$\begin{gathered} \left[ {\begin{array}{*{20}c} {\begin{array}{*{20}c} {var\left( {{\mathbf{g}}_{\mathbf{y}} } \right)} & {cov\left( {{\mathbf{g}}_{\mathbf{y}} ,{\mathbf{g}}_{{\mathbf{y}_{{\user2{g \times e}}} }} } \right)} & {cov\left( {{\mathbf{g}}_{\mathbf{y}} ,{\mathbf{g}}_{\user2{c}} } \right)} \\ {cov\left( {{\mathbf{g}}_{\mathbf{y}} ,{\mathbf{g}}_{{\mathbf{y}_{{\user2{g \times e}}} }} } \right)\user2{~}} & {var\left( {{\mathbf{g}}_{{\mathbf{y}_{{\user2{g \times e}}} }} } \right)} & {cov\left( {{\mathbf{g}}_{{\mathbf{y}_{{\user2{g \times e}}} }}, {\mathbf{g}}_{\user2{c}} } \right)} \\ {cov\left( {{\mathbf{g}}_{\mathbf{y}}, {\mathbf{g}}_{\user2{c}} } \right)} & {cov\left( {{\mathbf{g}}_{{\mathbf{y}_{{\user2{g \times e}}} }}, {\mathbf{g}}_{\user2{c}} } \right)} & {var\left( {{\mathbf{g}}_{\user2{c}} } \right)} \\ \end{array} } \\ \end{array} } \right] = \hfill \\ \left[ {\begin{array}{*{20}c} {\begin{array}{*{20}c} {0.4} & 0 & {0,~0.179,~0.358} \\ 0 & 0 & 0 \\ {0,~0.179,~0.358} & 0 & {0.5} \\ \end{array} } \\ \end{array} } \right] \hfill \\ \end{gathered}$$

The covariance term, $\:cov({\mathbf{g}}_{\mathbf{y}}\:,{\mathbf{g}}_{\boldsymbol{c}})$, resulted in genetic correlations of 0, 0.4 and 0.8, represents a realistic range of genetic correlation between two traits (**y** and **c**).

Additionally, we incorporated the main effect of the exposure (e.g. $\:{\beta\:}_{c}$= 0.447), accounting for 20% of the total phenotypic variance in the outcome. In this case, the variance-covariance structure was slightly modified as follows:

$$\begin{gathered} \left[ {\begin{array}{*{20}c} {\begin{array}{*{20}c} {var\left( {\boldsymbol{\beta }_{{{\mathbf{add}}}} } \right)} & {cov\left( {\boldsymbol{\beta }_{{{\mathbf{add}}}} ,\boldsymbol{\beta }_{{\mathbf{g \times e}}} } \right)} & {cov\left( {\boldsymbol{\beta }_{{{\mathbf{add}}}} ,\boldsymbol{\beta }_{{{\mathbf{add}}_{{\mathbf{c}}} ~}} } \right)} \\ {cov\left( {\boldsymbol{\beta }_{{{\mathbf{add}}}} ,\boldsymbol{\beta }_{{\mathbf{g \times e}}} } \right)\user2{~}} & {var\left( {\boldsymbol{\beta }_{{\mathbf{g \times e}}} } \right)} & {cov\left( {\boldsymbol{\beta }_{{{\mathbf{add}}_{{\mathbf{c}}} ~}} ,\boldsymbol{\beta }_{{\mathbf{g \times e}}} } \right)} \\ {cov\left( {\boldsymbol{\beta }_{{{\mathbf{add}}}} ,\boldsymbol{\beta }_{{{\mathbf{add}}_{{\mathbf{c}}} ~}} } \right)} & {cov\left( {\boldsymbol{\beta }_{{{\mathbf{add}}_{{\mathbf{c}}} ~}} ,\boldsymbol{\beta }_{{\mathbf{g \times e}}} } \right)} & {var\left( {\boldsymbol{\beta }_{{{\mathbf{add}}_{{\mathbf{c}}} ~}} } \right)} \\ \end{array} } \\ \end{array} } \right] \hfill \\ = \left[ {\begin{array}{*{20}c} {\begin{array}{*{20}c} {0.2} & 0 & {0, 0.126, 0.253} \\ 0 & 0 & 0 \\ {0, 0.126, 0.253} & 0 & {0.5} \\ \end{array} } \\ \end{array} } \right] \hfill \\ \end{gathered}$$

The residual components, **ε**_**y**_, $\boldsymbol{\varepsilon }_{{{\mathbf{y}}_{{{\mathbf{r \times e}}}} }} \:$and **ε**_**c**_ were simulated as multivariate normal random variables. In addition to the residual components $\boldsymbol{\varepsilon }_{\mathbf{y}}$ and **ε**_**c**_ defined in Eqs. 1 and 4, $\boldsymbol{\varepsilon }_{{{\mathbf{y}}_{{{\mathbf{r \times e}}}} }}$ is a newly introduced in this simulation setting. This term represents residual-by-environment interactions, introducing residual heteroscedasticity, which leads to heterogenous residual variance across different environmental levels. Such residual heteroscedasticity can result in model misspecification if not explicitly accounted for, making it a key source of spurious G×E signals. Initially, we conducted simulations without residual-by-environment interactions, setting var $\left( {\boldsymbol{\varepsilon }_{{{\mathbf{y}}_{{{\mathbf{r \times e}}}} }} } \right) = 0$. In this case, the residual components were simulated with a mean of zero and variances defined by the following variance-covariance matrix:

$$\begin{gathered} \left[ {\begin{array}{*{20}c} {\begin{array}{*{20}c} {var\left( {{\boldsymbol{\varepsilon }}_{{\mathbf{y}}} } \right)} & {cov\left( {{\boldsymbol{\varepsilon }}_{{\mathbf{y}}} ,{\boldsymbol{\varepsilon }}_{{{\mathbf{y}}_{{{\mathbf{r \times e}}}} }} } \right)} & {cov\left( {{\boldsymbol{\varepsilon }}_{{\mathbf{y}}} ,{\boldsymbol{\varepsilon }}_{{\mathbf{c}}} } \right)} \\ {cov\left( {{\boldsymbol{\varepsilon }}_{{\mathbf{y}}} ,{\boldsymbol{\varepsilon }}_{{{\mathbf{y}}_{{{\mathbf{r \times e}}}} }} } \right)~} & {var\left( {{\boldsymbol{\varepsilon }}_{{{\mathbf{y}}_{{{\mathbf{r \times e}}}} }} } \right)} & {cov\left( {{\boldsymbol{\varepsilon }}_{{{\mathbf{y}}_{{{\mathbf{r \times e}}}} }} ,{\boldsymbol{\varepsilon }}_{{\mathbf{c}}} } \right)~} \\ {cov\left( {{\boldsymbol{\varepsilon }}_{{\mathbf{y}}} ,{\boldsymbol{\varepsilon }}_{{\mathbf{c}}} } \right)} & {cov\left( {{\boldsymbol{\varepsilon }}_{{{\mathbf{y}}_{{{\mathbf{r \times e}}}} }} ,{\boldsymbol{\varepsilon }}_{{\mathbf{c}}} } \right)~} & {var\left( {{\boldsymbol{\varepsilon }}_{{\mathbf{c}}} } \right)} \\ \end{array} } \\ \end{array} } \right] \hfill \\ = \left[ {\begin{array}{*{20}c} {\begin{array}{*{20}c} {0.6} & 0 & {0,~0.219,~0.438} \\ 0 & 0 & 0 \\ {0,~0.219,~0.438} & 0 & {0.5} \\ \end{array} } \\ \end{array} } \right] \hfill \\ \end{gathered} $$

Next, we introduced residual-by-environment interactions by setting var ${\mathrm{(}}\boldsymbol{\varepsilon }_{{{\mathbf{y}}_{{{\mathbf{r \times e}}}} }} ) = 0.5$. The residual components were then simulated using the following variance-covariance matrix:

$$\begin{gathered} \left[ {\begin{array}{*{20}c} {\begin{array}{*{20}c} {var\left( {{\boldsymbol{\varepsilon }}_{{\mathbf{y}}} } \right)} & {cov\left( {{\boldsymbol{\varepsilon }}_{{\mathbf{y}}} ,{\boldsymbol{\varepsilon }}_{{{\mathbf{y}}_{{{\mathbf{r \times e}}}} }} } \right)} & {cov\left( {{\mathbf{\varepsilon }}_{{\mathbf{y}}} ,{\boldsymbol{\varepsilon }}_{{\mathbf{c}}} } \right)} \\ {cov\left( {{\boldsymbol{\varepsilon }}_{{\mathbf{y}}} ,{\boldsymbol{\varepsilon }}_{{{\mathbf{y}}_{{{\mathbf{r \times e}}}} }} } \right)~} & {var\left( {{\boldsymbol{\varepsilon }}_{{{\mathbf{y}}_{{{\mathbf{r \times e}}}} }} } \right)} & {cov\left( {{\boldsymbol{\varepsilon }}_{{{\mathbf{y}}_{{{\mathbf{r \times e}}}} }} ,{\boldsymbol{\varepsilon }}_{{\mathbf{c}}} } \right)~} \\ {cov\left( {{\boldsymbol{\varepsilon }}_{{\mathbf{y}}} ,{\mathbf{\varepsilon }}_{{\mathbf{c}}} } \right)} & {cov\left( {{\boldsymbol{\varepsilon }}_{{{\mathbf{y}}_{{{\mathbf{r \times e}}}} }} ,{\boldsymbol{\varepsilon }}_{{\mathbf{c}}} } \right)~} & {var\left( {{\boldsymbol{\varepsilon }}_{{\mathbf{c}}} } \right)} \\ \end{array} } \\ \end{array} } \right] \hfill \\ = \left[ {\begin{array}{*{20}c} {\begin{array}{*{20}c} {0.1} & 0 & {0,~0.089,~0.179} \\ 0 & {0.5} & 0 \\ {0,~0.089,~0.179} & 0 & {0.5} \\ \end{array} } \\ \end{array} } \right] \hfill \\ \end{gathered}$$

These simulations maintained genetic variance values of 0.4 for the outcome and 0.5 for the exposure variable for data simulated without the main effects of exposure ($\:{\beta\:}_{c}=0$). For the data simulated with main effects of exposure ($\:{\beta\:}_{c}=0.447$), the contribution of genetic variance was set to 0.2 for the outcome. These simulation settings ensured realistic genetic structure while assessing the impact of residual heteroscedasticity. The covariance term, $\:cov(\boldsymbol{\varepsilon }_{{\mathbf{y}}} ,\boldsymbol{\varepsilon }_{{\mathbf{c}}} )$, resulted in residual correlations of 0, 0.4 and 0.8, represents a realistic range of residual correlation between two traits (**y** and **c**).

Using simulated phenotypic data for **y** and c under the null model for G×E across various scenarios, we applied Models 1–4 with both correct and reverse causal directions to assess whether any spurious G×E signals emerged in either direction.

#### Simulating the alternative model for GxE

In the alternative model, the data were simulated to include GxE (var(**β**_**gxe**_) = 0.1). Consequently, the variance-covariance matrix was modified as follows:

$$\begin{gathered} \left[ {\begin{array}{*{20}c} {\begin{array}{*{20}c} {var\left( {\boldsymbol{\beta }_{{{\mathbf{add}}}} } \right)} & {cov\left( {\boldsymbol{\beta }_{{{\mathbf{add}}}} ,\boldsymbol{\beta }_{{\mathbf{g \times e}}} } \right)} & {cov\left( {\boldsymbol{\beta }_{{{\mathbf{add}}}} ,\boldsymbol{\beta }_{{{\mathbf{add}}_{{\mathbf{c}}} ~}} } \right)} \\ {cov\left( {\boldsymbol{\beta }_{{{\mathbf{add}}}} ,\boldsymbol{\beta }_{{\mathbf{g \times e}}} } \right)\user2{~}} & {var\left( {\boldsymbol{\beta }_{{\mathbf{g \times e}}} } \right)} & {cov\left( {\boldsymbol{\beta }_{{{\mathbf{add}}_{{\mathbf{c}}} ~}} ,\boldsymbol{\beta }_{{\mathbf{g \times e}}} } \right)} \\ {cov\left( {\boldsymbol{\beta }_{{{\mathbf{add}}}} ,\boldsymbol{\beta }_{{{\mathbf{add}}_{{\mathbf{c}}} ~}} } \right)} & {cov\left( {\boldsymbol{\beta }_{{{\mathbf{add}}_{{\mathbf{c}}} ~}} ,\boldsymbol{\beta }_{{\mathbf{g \times e}}} } \right)} & {var\left( {\boldsymbol{\beta }_{{{\mathbf{add}}_{{\mathbf{c}}} ~}} } \right)} \\ \end{array} } \\ \end{array} } \right] \hfill \\ = \left[ {\begin{array}{*{20}c} {\begin{array}{*{20}c} {0.3} & 0 & {0,~0.155,~0.31} \\ 0 & {0.1} & 0 \\ {0,~0.155,~0.31} & 0 & {0.5} \\ \end{array} } \\ \end{array} } \right] \hfill \\ \end{gathered} $$

In this alternative model, we also incorporated the main effect of the exposure (e.g. $\:{\beta\:}_{c}$= 0.447), accounting for 20% of the total phenotypic variance in the outcome. This led to a slight modification of the variance-covariance structure:

$$\begin{gathered} \left[ {\begin{array}{*{20}c} {\begin{array}{*{20}c} {var\left( {{\boldsymbol{\beta }}_{{{\mathbf{add}}}} } \right)} & {cov\left( {{\boldsymbol{\beta }}_{{{\mathbf{add}}}} ,{\boldsymbol{\beta }}_{{\mathbf{g \times e}}} } \right)} & {cov\left( {{\boldsymbol{\beta }}_{{{\mathbf{add}}}} ,{\boldsymbol{\beta }}_{{{\mathbf{add}}_{{\mathbf{c}}} ~}} } \right)} \\ {cov\left( {{\boldsymbol{\beta }}_{{{\mathbf{add}}}} ,{\boldsymbol{\beta }}_{{\mathbf{g \times e}}} } \right)\user2{~}} & {var\left( {{\boldsymbol{\beta }}_{{\mathbf{g \times e}}} } \right)} & {cov\left( {{\boldsymbol{\beta }}_{{{\mathbf{add}}_{{\mathbf{c}}} ~}} ,{\boldsymbol{\beta }}_{{\mathbf{g \times e}}} } \right)} \\ {cov\left( {{\boldsymbol{\beta }}_{{{\mathbf{add}}}} ,{\boldsymbol{\beta }}_{{{\mathbf{add}}_{{\mathbf{c}}} ~}} } \right)} & {cov\left( {{\boldsymbol{\beta }}_{{{\mathbf{add}}_{{\mathbf{c}}} ~}} ,{\boldsymbol{\beta }}_{{\mathbf{g \times e}}} } \right)} & {var\left( {{\boldsymbol{\beta }}_{{{\mathbf{add}}_{{\mathbf{c}}} ~}} } \right)} \\ \end{array} } \\ \end{array} } \right] \hfill \\ = \left[ {\begin{array}{*{20}c} {\begin{array}{*{20}c} {0.1} & 0 & {0,~0.089,~0.179} \\ 0 & {0.1} & 0 \\ {0,~0.089,~0.179} & 0 & {0.5} \\ \end{array} } \\ \end{array} } \right] \hfill \\ \end{gathered}$$

These simulation scenarios allow us to assess the impact of both G×E and the main effect of exposure on phenotypic variance while testing the causal directions using Models 1–4. The residual effects were simulated using the same approach as the null simulation described in Sect. Simulating the null model for GxE.

#### Simulating binary variables

The simulation methods described above were designed for quantitative outcome and exposure variables. To simulate binary outcomes and exposures, we transformed the quantitative data by designating the top 10th percentile as cases, resulting in a prevalence of 10%, with the remaining observations classified as controls. This transformation was applied to each simulation scenario to test scenarios where the outcome is binary, the exposure is quantitative, or both the outcome and exposure are binary.

### Real data analyses

To investigate the causal directions of GxE between anthropometric traits and circulating biomarkers, we employed a comprehensive analytical pipeline using UK Biobank data from 288,792 individuals. The cohort was divided into two independent, non-overlapping samples: a discovery sample (*n* = 231,034) for genome-wide association analyses and a target sample (*n* = 57,758) for causal direction assessment of GxE interactions. We examined three anthropometric traits (BMI, body fat percentage (BF), and waist-to-hip ratio (WHR) against eleven circulating biomarkers (alanine aminotransferase (ALT), aspartate aminotransferase (AST), apolipoprotein A (ApoA), apolipoprotein B (ApoB), total cholesterol, C-reactive protein (CRP), high-density lipoprotein cholesterol (HDL-c), low-density lipoprotein cholesterol (LDL-c), total bilirubin, triglycerides, and vitamin D). All genetic association analyses were adjusted for population stratification using the first 10 principal components, along with demographic (age, sex), socioeconomic (Townsend deprivation index), and technical covariates (batch effects and assessment centre) (Abegaz et al. 2019; Kaufman et al. 2004; Keller 2014). To establish causal direction, we conducted GWEIS in the discovery sample, treating each variable alternately as exposure and outcome. We also conducted GWAS in the discovery sample, treating each variable alternately as exposure based on the assigned roles. PRS were computed for both main genetic effects and interaction effects from GWEIS of the outcome, as well as additive genetic effects from GWAS of the exposure, using identified variants and then applied to the target sample using Plink2 software (Purcell et al. 2007). The causal direction analyses involved the four statistical models (the same Model 1–4, as used in the simulation validation) for each trait-biomarker pair. We first tested anthropometric traits as outcomes with biomarkers as exposures, then reversed the roles to test biomarkers as outcomes with anthropometric traits as exposures. This GxE causal direction approach (GCIM) applied to the target samples enabled a robust assessment of causal directions of GxE while controlling for confounding factors using the 15 covariates described above.

## Results

### Simulation results for quantitative traits

**Fig. 1:**
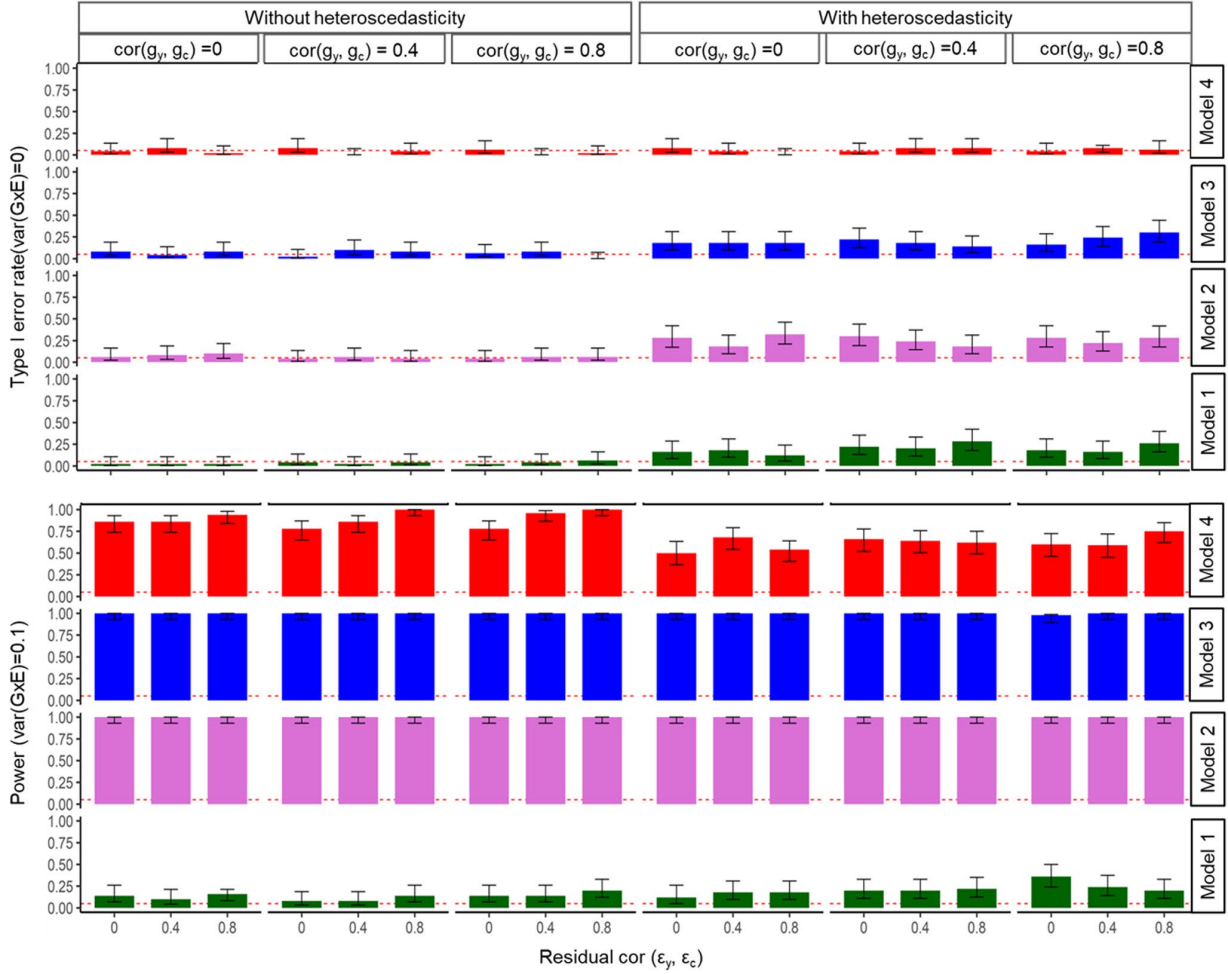
Simulation results for quantitative outcome and quantitative exposure under true causal direction. Results are shown across varying genetic correlations and residual correlations, under scenarios with and without heteroscedasticity (i.e. residual-by-environment interactions). The top panel reports type I error rates under the null, and the bottom panel shows power under the alternative. Within each panel, bars are grouped by genetic correlation cor$\:({\mathbf{g}}_{{\mathbf{y}}},{\mathbf{g}}_{\mathbf{c}})$= 0, 0.4, 0.8. For each genetic correlation, the three bars correspond to residual correlations of cor$(\boldsymbol{\varepsilon }_{{\mathbf{y}}} ,\boldsymbol{\varepsilon }_{{\mathbf{c}}} )$ = 0, 0.4, and 0.8. This structure illustrates how correlation and heteroscedasticity jointly affect method performance. Model 4 represents the proposed model (GCIM), whereas Models 1–3 correspond to existing methods

Following the simulation scenarios described above, we analysed the simulated datasets using Models 1 through 4 to infer the true direction of causation between **y** (outcome) and **c** (exposure). In the null simulation scenario i.e., with no true G×E and no residual-by-environment interactions, all four models (Models 1–4) correctly controlled the type I error rate for GxE (Fig. 1). However, when residual-by-environment interactions variance was set to 0.5, Models 1, 2, and 3 produced false-positive associations. In contrast, the GCIM method (Model 4) correctly identified the absence of G×E variance, avoiding spurious findings. When the G×E variance was increased to 0.1, all methods, except for Model 1 demonstrated adequate power to detect associations, irrespective of the residual-by-environment interactions. Model 1, however, showed substantially lower power (Fig. 1).

**Fig. 2:**
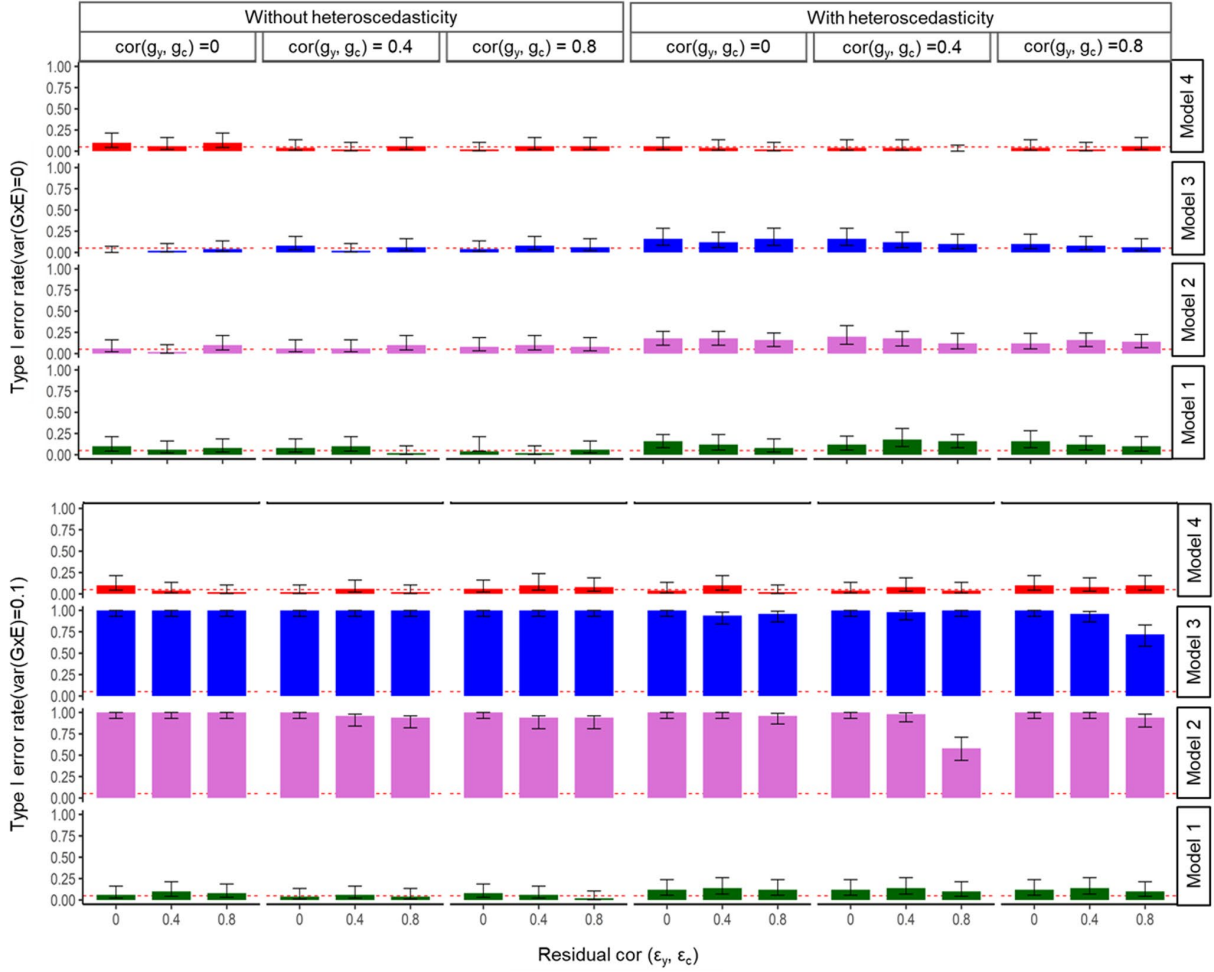
Simulation results for quantitative outcome and quantitative exposure under reverse causal direction. Results are shown across varying genetic correlations and residual correlations, under scenarios with and without heteroscedasticity (i.e. residual-by-environment interactions). The top panel reports type I error rates under the null, and the bottom panel shows type 1 error rate under the alternative for the reverse directions. Within each panel, bars are grouped by genetic correlation cor$\:({\mathbf{g}}_{{\mathbf{y}}},{\mathbf{g}}_{\mathbf{c}})$= 0, 0.4, 0.8. For each genetic correlation, the three bars correspond to residual correlations of cor$(\boldsymbol{\varepsilon }_{{\mathbf{y}}} ,\boldsymbol{\varepsilon }_{{\mathbf{c}}} )$ = 0, 0.4, and 0.8. This structure illustrates how correlation and heteroscedasticity jointly affect method performance. Model 4 represents the proposed model (GCIM), whereas Models 1–3 correspond to existing methods

To evaluate the reverse causal direction, treating **c** as the outcome and **y** as the exposure we conducted a similar analysis (Fig. 2). In the absence of residual-by-environment interactions, all models (Models 1–4) again appropriately controlled the type I error rate, as observed in the correct causal direction. However, when residual-by-environment interactions were introduced, Models 1, 2, and 3 yielded spurious G×E associations in the reverse causal direction. In contrast, the GCIM method (Model 4) maintained robustness and accurately detected the absence of G×E variance, avoiding false conclusions. When true G×E were present (var(G×E) = 0.1; Fig. 2), Model 1 appeared to control the type I error rate but generated spurious results when residual-by-environment interactions variance increased to 0.5. Similarly, Models 2 and 3 consistently produced spurious associations under reverse causality, regardless of the residual-by-environment interactions level. In contrast, the GCIM method effectively controlled spurious associations and demonstrated strong performance in distinguishing true G×E from false positives in the reverse causal direction (Fig. 2).

### Simulation results for binary traits with quantitative exposure

**Fig. 3:**
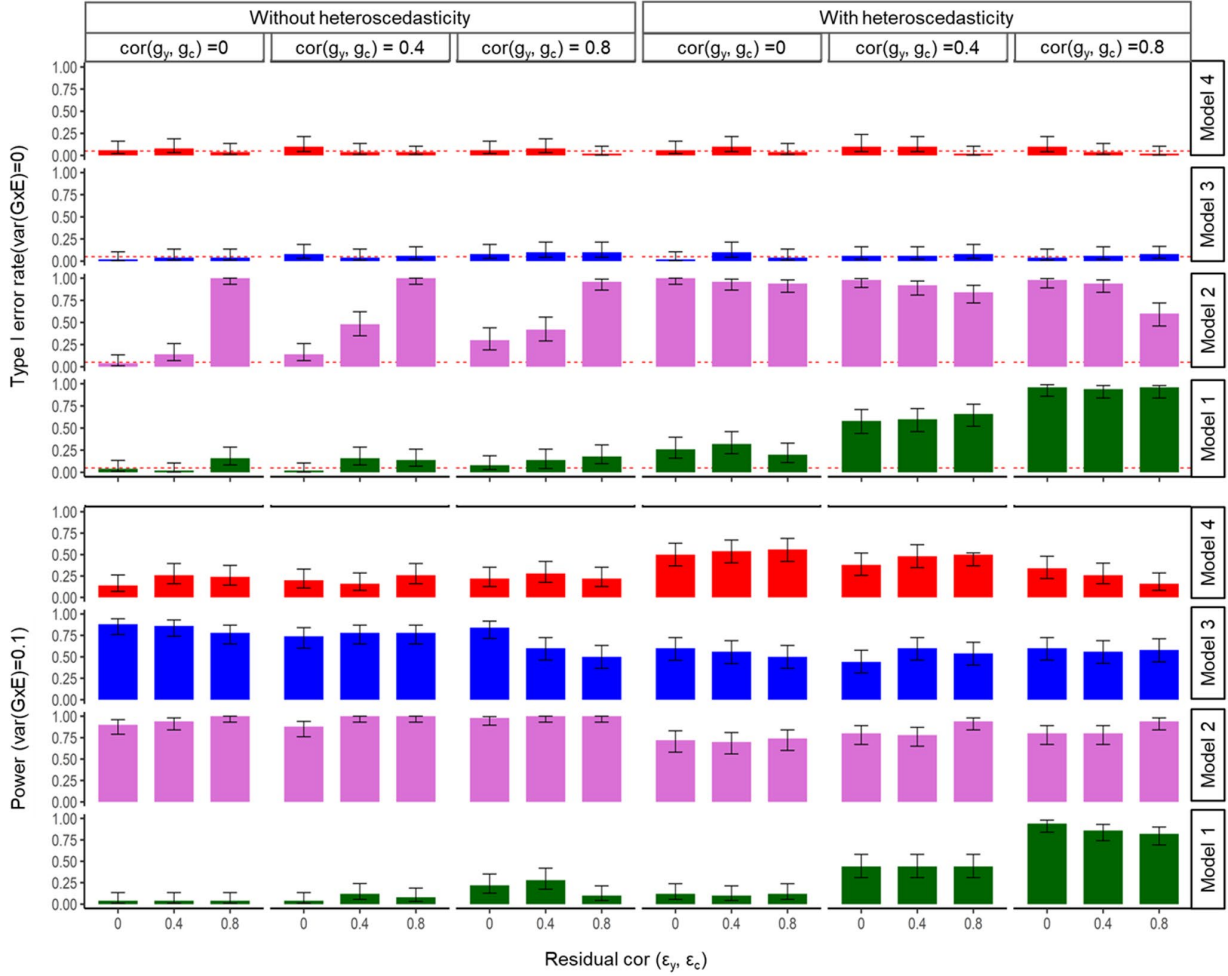
Simulation results for binary outcome and quantitative exposure under true causal direction. Results are shown across varying genetic correlations and residual correlations, under scenarios with and without heteroscedasticity (i.e. residual-by-environment interactions). The top panel reports type I error rates under the null, and the bottom panel shows power under the alternative. Within each panel, bars are grouped by genetic correlation cor$\:({\mathbf{g}}_{{\mathbf{y}}},{\mathbf{g}}_{\mathbf{c}})$= 0, 0.4, 0.8. For each genetic correlation, the three bars correspond to residual correlations of cor$(\boldsymbol{\varepsilon }_{{\mathbf{y}}} ,\boldsymbol{\varepsilon }_{{\mathbf{c}}} )$ = 0, 0.4, and 0.8. This structure illustrates how correlation and heteroscedasticity jointly affect method performance. Model 4 represents the proposed model (GCIM), whereas Models 1–3 correspond to existing methods

In simulations involving binary outcomes (with a 10% case prevalence) and a quantitative exposure, Models 1 and 2 produced false-positive G×E associations under the null model when residual-by-environment interactions were present (Fig. 3). Notably, Model 2 also generated spurious associations even in the absence of such interactions, indicating poor specificity. In contrast, both Model 3 and the GCIM method demonstrated robustness, correctly identifying the absence of G×E effects and avoiding false positives. Under the alternative model with a G×E variance of 0.1, Model 1 exhibited no power when residual-by-environment interactions were absent. In comparison, Models 2 and 3 achieved adequate power to detect G×E effects. However, GCIM showed limited power in this setting. When the residual-by-environment interactions variance increased to 0.5, Model 1 consistently demonstrated low power across all levels of genetic and residual correlations. These findings, summarized in Fig. 3, illustrate the varying effectiveness of each method under different simulation scenarios.

**Fig. 4:**
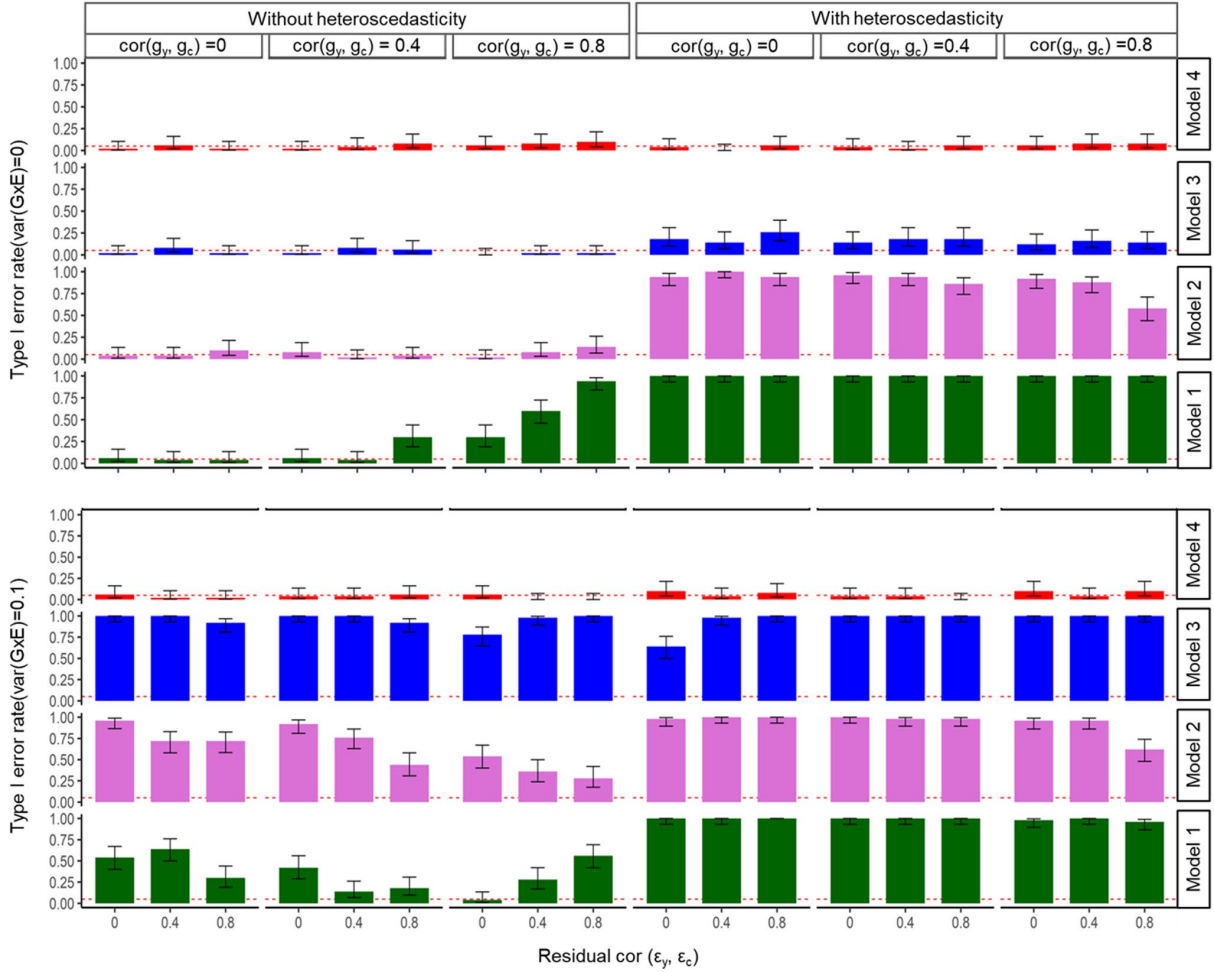
Simulation results for binary outcome and quantitative exposure under reverse causal direction. Results are shown across varying genetic correlations and residual correlations, under scenarios with and without heteroscedasticity (i.e. residual-by-environment interactions). The top panel reports type I error rates under the null, and the bottom panel shows type 1 error rate under the alternative for the reverse directions. Within each panel, bars are grouped by genetic correlation cor$\:({\mathbf{g}}_{{\mathbf{y}}}, {\mathbf{g}}_{\mathbf{c}})$= 0, 0.4, 0.8. For each genetic correlation, the three bars correspond to residual correlations of cor$(\boldsymbol{\varepsilon }_{{\mathbf{y}}} ,\boldsymbol{\varepsilon }_{{\mathbf{c}}} )$ = 0, 0.4, and 0.8. This structure illustrates how correlation and heteroscedasticity jointly affect method performance. Model 4 represents the proposed model (GCIM), whereas Models 1–3 correspond to existing methods

In the same simulation scenario but testing the reverse causal direction (with binary **y** as the exposure and quantitative **c** as the outcome), data simulated under the null model with no residual-by-environment interactions still showed spurious G×E signals in both Model 1 and Model 2, indicating a lack of specificity (Fig. 4). When residual-by-environment interaction variance was introduced under the null model, all three models Model 1, Model 2, and Model 3 consistently produced false-positive G×E associations in the reverse causal direction. In contrast, the GCIM method accurately identified the absence of G×E variance, avoiding spurious results in all cases. Under the alternative model, where G×E variance was set to 0.1, Models 1–3 continued to generate spurious associations in the reverse causal direction, regardless of whether residual-by-environment interactions were present. Meanwhile, the GCIM method remained robust, accurately distinguishing true from false associations in both null and alternative models, across all interaction settings. As shown in Fig. 4, GCIM consistently controlled false-positive G×E signals and demonstrated strong performance in handling reverse causality scenarios.

### Simulation results for binary outcome with binary exposure

In the null model simulations, both Model 1 and Model 2 produced spurious G×E associations, failing to detect the absence of G×E variance. In contrast, Model 3 and the GCIM method correctly control the type 1 error rate (Fig S2). In the alternative model simulations, most methods, including Model 1, Model 3, and GCIM, exhibited low power. However, Model 2 demonstrated better power than the other methods across most scenarios, including those with varying genetic and residual correlations, both with and without residual-by-environment interactions. As shown in Fig S2, while GCIM effectively controlled spurious associations in scenarios with no G×E variance, it had low power in detecting true G×E when the G×E variance was set to 0.1. In the same simulation scenario, testing the reverse causal direction (with binary **y** as the exposure and binary **c** as the outcome), data simulated with both the absence of residual-by-environment interaction and with residual-by-environment interactions of 0.5 revealed that Model 1 and Model 2 produced spurious G×E associations in the reverse causal direction (Fig S3). In contrast, Model 3 and the GCIM methods accurately identified the absence of G×E, avoiding spurious conclusions. For datasets simulated under the alternative model, Model 1, Model 2, and Model 3 consistently generated spurious associations in the reverse causal direction, regardless of the presence of residual-by-environment interactions. However, the GCIM method showed robust performance, effectively avoiding spurious associations in both scenarios whether there were no residual-by-environment interactions or interactions of 0.5 across all genetic and residual correlation settings. As shown in Fig S3, the GCIM method consistently controlled spurious G×E associations and demonstrated superior ability to distinguish true associations from spurious ones in the reverse causal direction.

The performance of GCIM in controlling the Type 1 error rate for binary outcomes with binary exposures was assessed across multiple scenarios, varying the level of GxE variance or the prevalence of an outcome. When the GxE variance was set to zero (null model), the empirical type 1 error rate remained close to 0.05, indicating appropriate control. As the GxE variance increased up to 0.3, the power of detecting GxE rose significantly above 0.05, reflecting true signal detection rather than inflated error. Additionally, higher outcome prevalence in the presence of GxE led to increased power, highlighting the model’s effectiveness in identifying true effects in more common outcomes. These results demonstrate that GCIM maintains adequate power across different levels of GxE variance and prevalence of cases (Fig S4).

Data were additionally simulated under null model that included the effects of exposure but tested without adjusting for the main effects of exposure. Under these conditions, model 3 produced spurious associations, especially when residual-by-environment interactions were high. In contrast, the proposed model effectively controlled for these spurious associations for both binary and quantitative traits (Figs. S5 and S6).

### Real data analyses

We conducted GxE analyses using four statistical models: Models 1–3 and the proposed Model 4 (GCIM), focusing on three anthropometric traits and eleven circulating biomarkers. To assess the causal directions of GxE, each anthropometric trait–biomarker pair was tested under two hypotheses: (1) genetic effects on the anthropometric trait are modified by biomarker levels, and (2) the reverse, where the anthropometric trait modifies genetic effects on the biomarker.

#### BMI

**Fig. 5:**
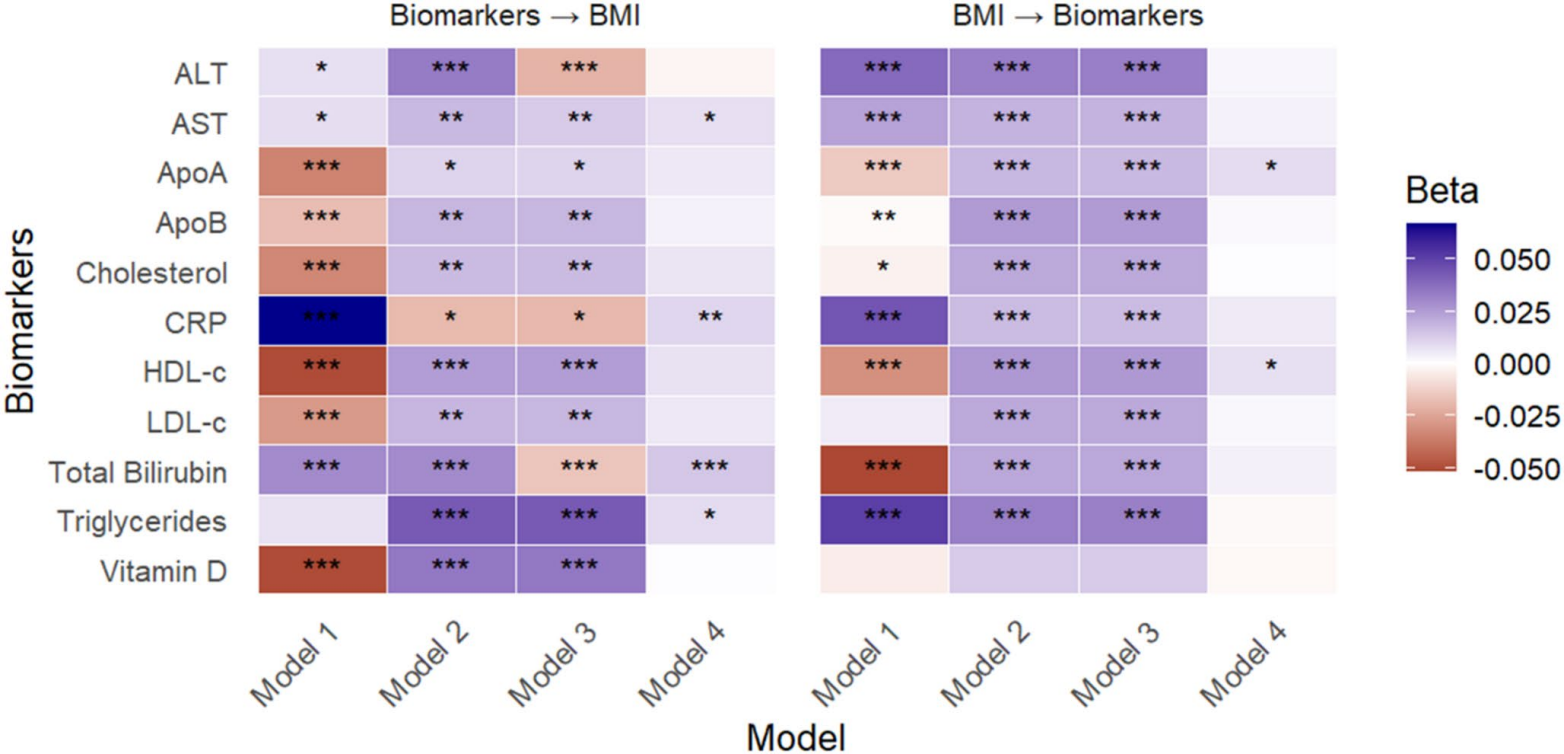
GxE analyses between BMI and biomarkers, evaluating BMI as the main outcome in the proposed (forward) direction and as the exposure in the reverse direction, using Models 1–4. * P-values < 0.05, ** P-values < 0.01, *** P-values < 0.001. Model 4 represents the proposed model (GCIM), whereas Models 1–3 correspond to existing methods

Comparisons between traditional GxE models (Models 1–3) and GCIM revealed substantial differences in interaction detection. Traditional models identified multiple significant GxE interactions involving BMI; however, the directionality of these interactions was indistinguishable, as significant signals were observed for both causal directions (Fig. 5). For several biomarkers such as AST, total bilirubin, CRP, and triglycerides, traditional methods reported significant interactions regardless of direction. In contrast, GCIM (Model 4) detected significant interactions only when these biomarkers were modelled as environmental modifier of genetic effects on BMI, indicating a clear causal direction from biomarker to BMI (Fig. 5).

For ApoA and HDL-c, traditional models again showed significant GxE effects in both directions, offering no insight into causal direction. However, GCIM revealed that BMI modifies the genetic effects on these biomarkers, suggesting a causal direction from BMI to ApoA and HDL-c (Fig. 5). After Bonferroni correction for multiple testing (study-wide threshold of 0.05/66), only the causal effect of bilirubin on BMI remained significant, providing robust evidence.

#### Body fat percentages

As expected, traditional G×E models (Models 1–3) identified widespread significant interactions between body fat percentage (BF) and various biomarkers in both causal directions, offering no insight into the true causal direction.

**Fig. 6:**
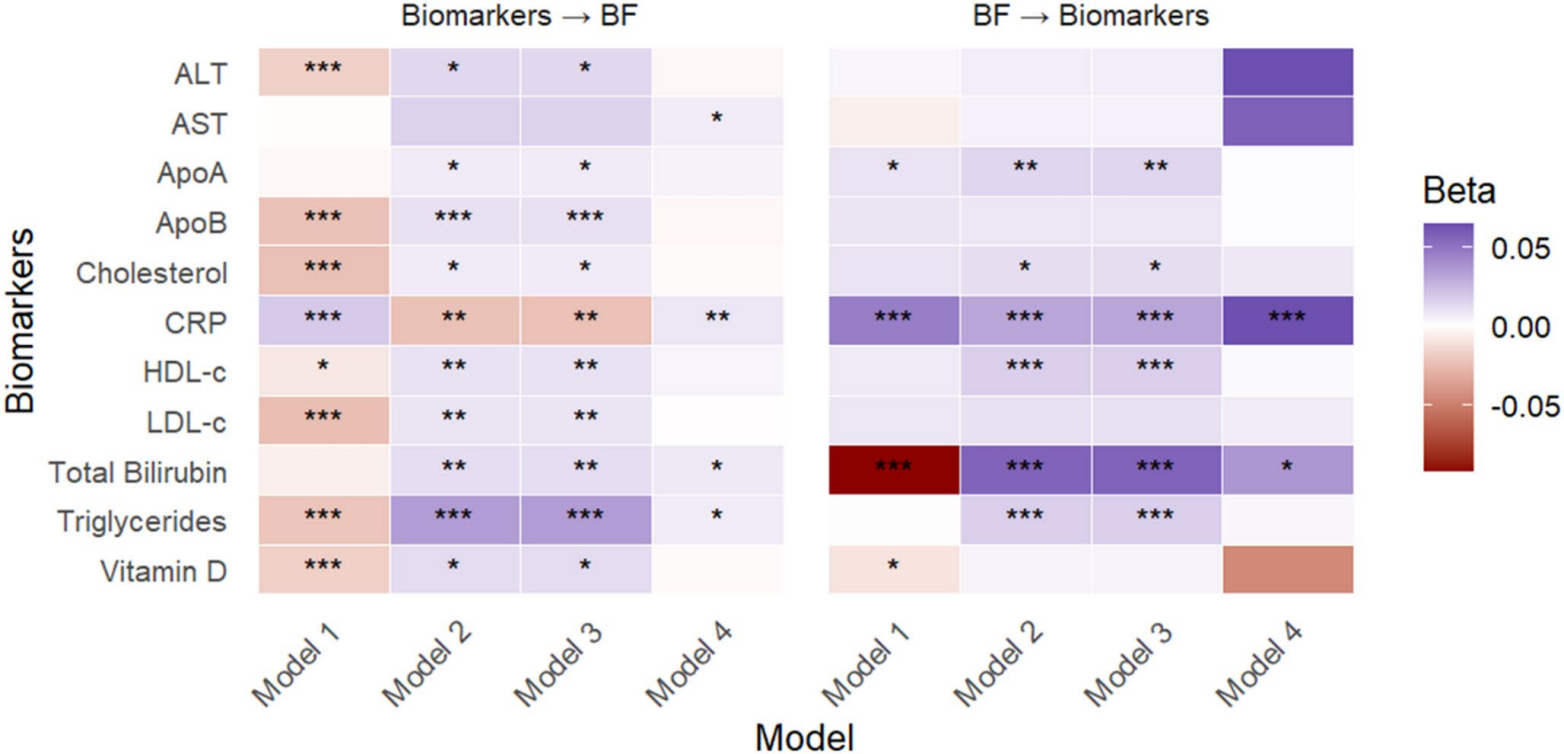
GxE analysis between BF and biomarkers, evaluating BF as the main outcome in the proposed (forward) direction and as the exposure in the reverse direction, using Models 1–4. * P-values < 0.05, ** P-values < 0.01, *** P-values < 0.001. Model 4 represents the proposed model (GCIM), whereas Models 1–3 correspond to existing methods

In contrast, for AST and triglyceride, GCIM (Model 4) detected significant interactions only when the biomarker was modelled as the environmental modifier of genetic effects on BF, indicating a clear causal direction from the biomarker to BF (Fig. 6). Additionally, GCIM revealed significant bidirectional G×E for CRP and bilirubin concentrations, where both causal directions were statistically significant (Fig. 6). However, after Bonferroni correction for multiple testing (study-wide threshold of 0.05/66), only the causal effect of BF on CRP remained significant.

#### Waist-to-hip ratio

**Fig. 7:**
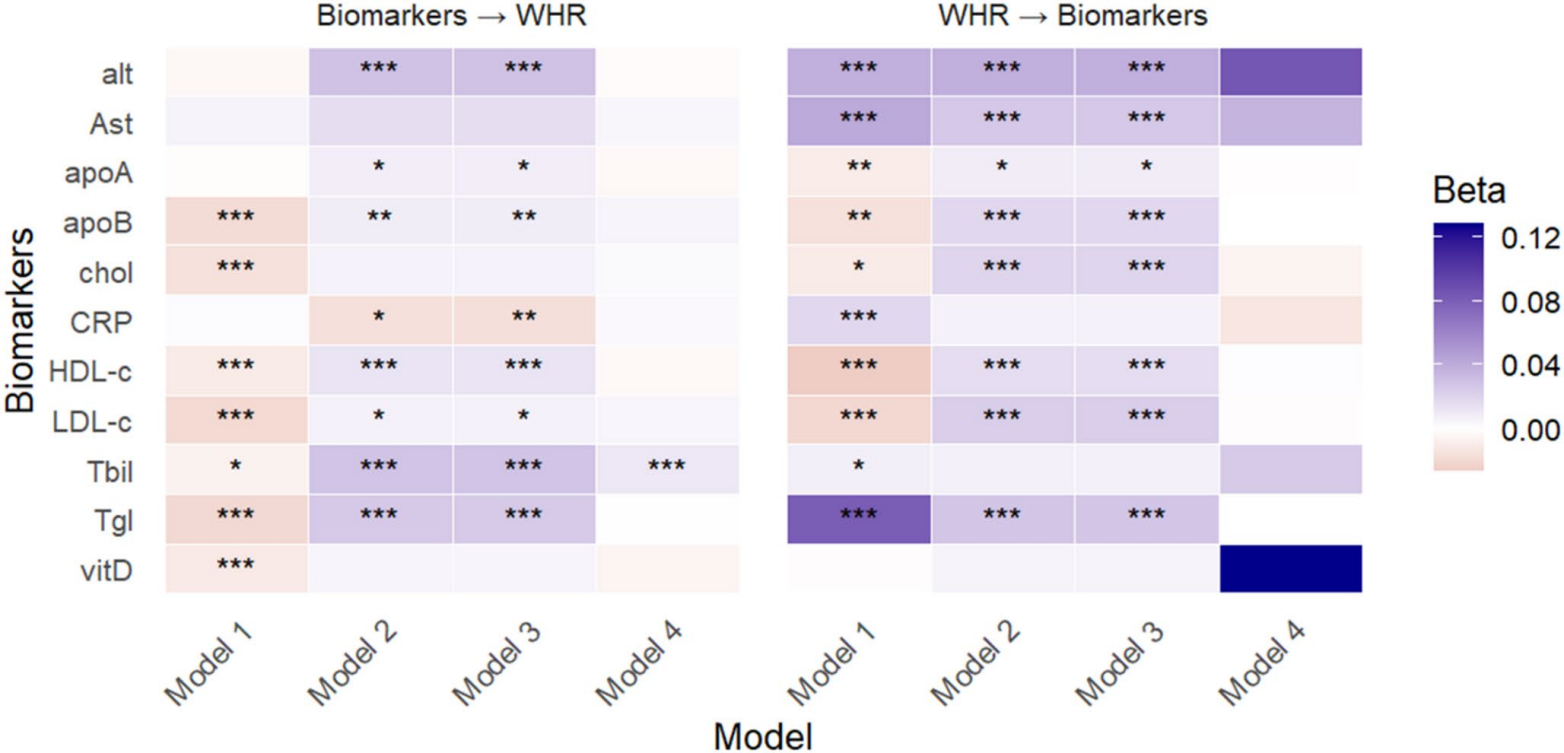
GxE analyses between WHR and biomarkers, evaluating WHR as the main outcome in the proposed (forward) direction and as the exposure in the reverse direction, using Models 1–4. * P-values < 0.05, ** P-values < 0.01, *** P-values < 0.001. Model 4 represents the proposed model (GCIM), whereas Models 1–3 correspond to existing methods

While traditional G×E models (Models 1–3) revealed multiple significant interactions between waist-to-hip ratio (WHR) and various biomarkers in both causal directions (Fig. 7), most of these signals were not supported by GCIM. This discrepancy may be due to either the absence of genuine causal effects or limited power of GCIM. GCIM identified significance only for the causal effect of bilirubin on WHR, consistent with the results from Models 2 and 3 (Fig. 7).

## Discussion

This study introduces and evaluates the GCIM framework as a robust approach for detecting G×E and inferring causal directions between environmental exposures and phenotypic outcomes. GCIM addresses several limitations of existing models, particularly their vulnerability to residual-by-environment interactions (i.e., heteroscedasticity), which can confound true G×E effects (White 1980). Our findings suggest that GCIM demonstrates improved robustness under such complex conditions, maintaining specificity where conventional models tend to produce spurious associations. This robustness is especially critical given the increasing interest in uncovering genuine biological interactions in structured and noisy datasets. Notably, GCIM’s consistent performance across different causal directions reinforces its practical value in applied research settings where reverse causality is a concern.

Our simulation studies revealed critical violations of statistical assumptions in traditional approaches. For quantitative traits, traditional methods (Models 1–3) exhibited inflated Type I error rates under conditions of residual heteroscedasticity, while GCIM maintained the nominal 5% false positive rate. The inflation of type 1error rate was also particularly severe for binary phenotypes, where Model 1 demonstrated elevated genetic and residual correlation parameters under heteroscedastic conditions. Most concerning was Model 2’s binary trait analyses, achieving 100% false positivity rates alongside increasing patterns of residual and genetic correlation. In contrast, Models 3 and 4 (GCIM) consistently maintained appropriate Type I error control at the expected 5% rate, demonstrating robust statistical properties across different trait distributions and genetic architectures. This substantial reduction in spurious associations addresses a critical challenge in contemporary statistical genetics: the multiple testing burden inherent in large-scale biobank studies where hundreds of trait-environment combinations undergo simultaneous testing, potentially preventing thousands of false discoveries that could misdirect genomic medicine research and clinical translation efforts.

Regarding statistical power, our findings are generally consistent with previous studies (Jayasinghe et al. 2024; Plomin et al. 2022; Tang et al. 2022), showing that Model 2 and Model 3 possess reasonable sensitivity in detecting true interactions, while Model 1 exhibits limited power. However, when the causal direction is misidentified, Model 2 and Model 3 become prone to inflated false positives. In contrast, GCIM appears to mitigate this issue effectively, demonstrating stability across both quantitative and binary outcomes. Specifically, by replacing exposure variable with PRS of exposure variable, GCIM​ mitigates spurious covariance and thereby reduces bias from shared residuals in the reverse model (see Supplementary note). However, conservative approach of GCIM to enhance robustness, it introduces limitations. Notably, our simulations indicate a reduction in statistical power, particularly for binary outcomes where GCIM detected fewer true interactions compared to Model 3. This power reduction stems from using PRS instead of observed phenotypes, which inherently captures less phenotypic variance. Moreover, GCIM provides an empirical test of directional asymmetry consistent with a causal interpretation under certain key assumptions, but it does not estimate a fully identified causal effect. As a result, it does not constitute a formal causal identification theorem under all data-generating mechanisms. Nevertheless, GCIM offers a conservative and reliable framework, especially well-suited to hypothesis-driven research or causal inference applications.

In our real data analysis exploring the relationship between anthropometric traits and biomarkers, GCIM revealed a novel directional finding: bilirubin significantly modulates the genetic effects on BMI, whereas the reverse direction is not supported by GCIM. While Models 1–3 detect significant interactions in both directions, GCIM’s directional result is more consistent with previous evidence (Gordon et al. 2021; Stec et al. 2016). Importantly, those earlier studies were conducted at the phenotypic level and did not directly model GxE. As such, they may only partially reflect underlying GxE effects. The findings from GCIM therefore offer a more robust and mechanistically informed interpretation. This is further supported by known biological pathways, as bilirubin has been shown to increase muscle mass and reduce fat accumulation, providing a protective effect against obesity (Jenko-Pražnikar et al. 2013; Kumagai et al. 2013; Wu et al. 2011). A similar pattern was observed for waist–hip ratio (WHR), where total bilirubin levels modulate the genetic effects on WHR. This finding is consistent with prior reports suggesting a role of bilirubin in regulating central adiposity (Gordon et al. 2021; Kipp et al. 2023; Stec et al. 2016), in line with its known anti-inflammatory and metabolic benefits.

We also found evidence that body fat modifies the genetic influence on CRP levels, highlighting a potential G×E mechanism underlying inflammatory regulation (Eiriksdottir et al. 2009; Li et al. 2023). This finding aligns with previous studies reporting adiposity gene interactions affecting CRP and systemic inflammation (Garske et al. 2023; Nienaber-Rousseau et al. 2014). The unique contribution of GCIM is clarifying the causal direction: supporting the interpretation that body fat modulates genetic effects on CRP, rather than the reverse. This is biologically plausible, as CRP is a downstream marker of inflammation and more likely influenced by adiposity status.

In summary, GCIM represents a meaningful advance in G×E methodology, particularly for studies, where controlling spurious associations and correctly inferring causal direction are important. Its robustness to confounding, model misspecification, and heteroscedasticity enhances confidence in detected signals. Nevertheless, its reduced power, particularly in binary outcome contexts, warrants further methodological refinement. Future work should aim to improve the sensitivity of GCIM without compromising its notable specificity and robustness.

### Software availability

The GCIM has been implemented as an open-source R package to facilitate reproducibility and broader application in GxE. Detailed documentation, example scripts, and tutorials are included to guide users in applying the methods to their own data. The GCIM R package is freely available at https://github.com/Zinabuf/GCIM, and can be installed directly in R.

## Supplementary Information

Below is the link to the electronic supplementary material.

Supplementary Material 1

## Data Availability

The data supporting the findings of this study are available through an approved data request from the UK Biobank, which can be accessed via their official website at https://www.ukbiobank.ac.uk/. The corresponding code are accessible through GitHub on https://github.com/Zinabuf/GCIM.
