# Supplementary material for: Leveraging polygenic risk scores to infer causal directions in genotype-by-environment interactions between complex traits
Source: Hum Genet. 2026 Feb 7;145(1):19. doi: 10.1007/s00439-025-02799-x (PMC12882956; doi:10.1007/s00439-025-02799-x)
Supplement: Supplementary file 1 — Supplementary Material 1 [file 439_2025_2799_MOESM1_ESM.docx]

**Leveraging polygenic risk scores to infer causal directions in genotype-by-environment interactions between complex traits.**

Authors: Zinabu Fentaw^1, 2, 3,4^, Buu Truong^5, 6^, Dovini Jayasinghe^1, 2, 3^, Chris Della Vedova ^7^, Gibran Hemani^8,9^, Beben Benyamin^1, 2, 3^, Elina Hyppönen^1, 3, 7^, S. Hong Lee^1, 2, 3^

Affiliations:

^1^Australian Centre for Precision Health, University of South Australia, Adelaide, South Australia, Australia

^2^UniSA Allied Health and Human Performance, University of South Australia, Adelaide, South Australia, Australia

^3^South Australian Health and Medical Research Institute (SAHMRI), University of South Australia, Adelaide, South Australia, Australia

^4^Department of Epidemiology and Biostatistics, School of Public Health, Wollo University, Dessie, Ethiopia

^5^Program in Medical and Population Genetics and the Cardiovascular Disease Initiative, Broad Institute of MIT and Harvard, 415 Main St, Cambridge, MA 02142.

^6^Center for Genomic Medicine and Cardiovascular Research Center, Massachusetts General Hospital, 185 Cambridge Street, Boston, MA, 02114

^7^UniSA Clinical & Health Sciences, University of South Australia, Adelaide, SA 5001, Australia

^8^Medical Research Council Integrative Epidemiology Unit, University of Bristol, Bristol, UK

^9^Population Health Sciences, Bristol Medical School, University of Bristol, Bristol, UK

*Correspondence: Zinabu Fentaw ([zinabu.werashe@mymail.unisa.edu.au](mailto:zinabu.werashe@mymail.unisa.edu.au)); S. Hong Lee ([hong.lee@unisa.edu.au](mailto:hong.lee@unisa.edu.au))

**Supplementary Note**

**Causal directions**

**
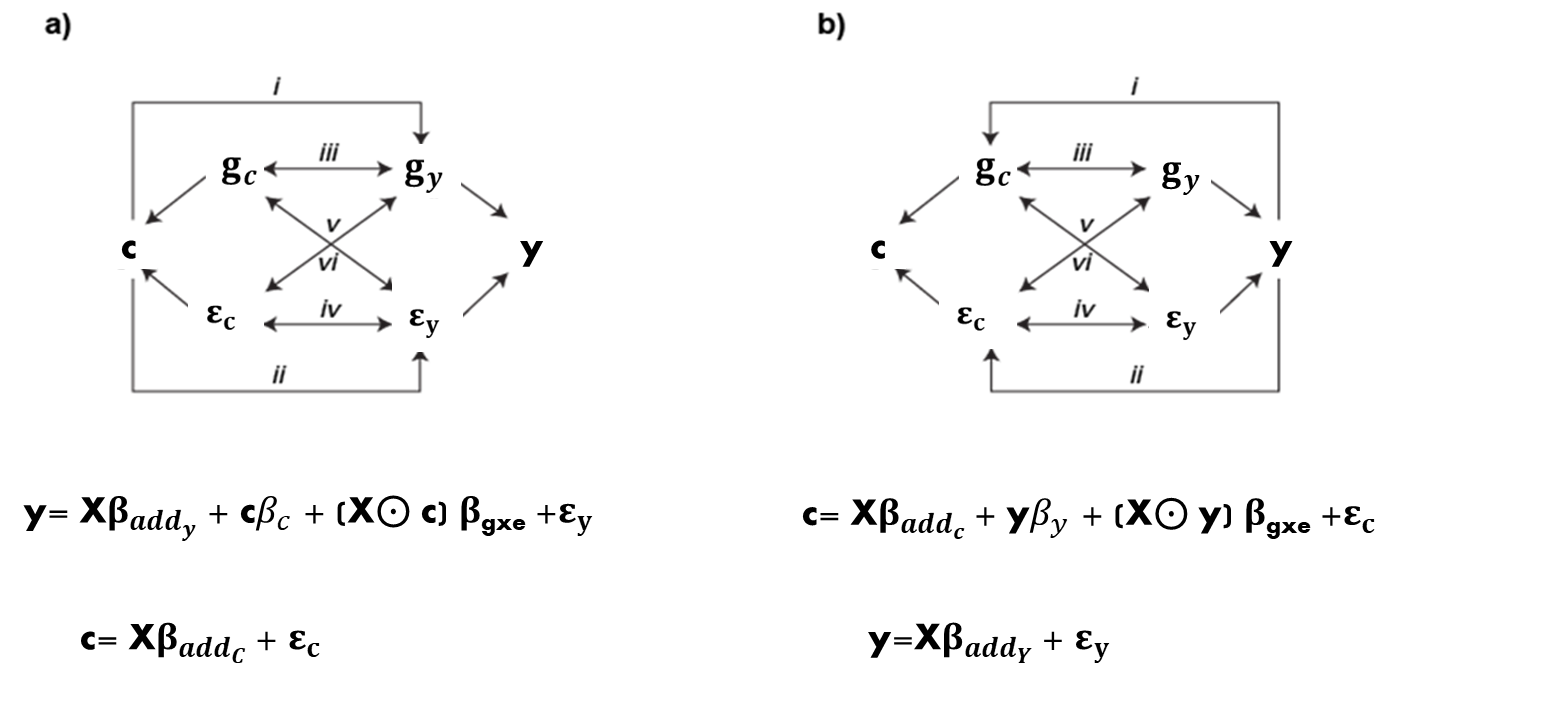
**

**Fig S1** The causal mechanism between exposure c and outcome y in the proposed (a) and reverse directions (b). In the proposed direction, the genetic effects of **y** (**g_y_**) are modulated by **c**. In the reverse direction, the genetic effects of **c** (**g_c_**) are modulated by Y. The terms, (**g_y_**)**,** (**g_c_**) and (**ε_y_**) (**ε_c_**) are genetic and non-genetic effects that are all possible effects underlying the phenotypes of outcome (exposure). Note that (**ε_y_**) (**ε_c_**) is any effects except (**g_y_**), (**g_c_**)**,** which include known and unknown confounding effects. Path i: the interaction of genetic effects of outcome to be modulated by exposure. Path ii: the interaction of non-genetic effects of outcome to be modulated by exposure. Path iii: Association between (**g_y_**) and (**g_c_**) because of pleiotropic effects (the same genes) or/and linkage disequilibrium (different genes). Path iv: Association between (**ε_y_**) and (**ε_c_**) because they share the same factors or have correlated factors. Path v: Association between (**g_c_**) and (**ε_y_**). Path vi: Association between (**g_c_**) and (**ε_c_**) Note that the association between **y** and **c** via the paths iii, iv, v and vi can be captured by the estimated regression coefficient, $\beta$, which is not the main parameter of interest in our random-effects GxE model, GCIM. Note that GCIM models the random effects of GxE after adjusting the association between **y** and **c**.

This section explains why existing GxE methods cannot be used to infer the causal directions. Assuming that the causal relationship involves only one direction, the correct direction can be determined by comparing models with proposed and reverse directions (Fig S1). However, when using observed outcome and exposure, the model with reverse direction can generate spurious interaction signals.

In the model with one direction (proposed direction), the model can be written as

**y =** $\mathbf{X}\boldsymbol{\beta}_{\boldsymbol{add}_{\boldsymbol{y}}}$ **+ c**$\beta_{c}$ **+** (**X⊙ c**) $\boldsymbol{\beta}_{\boldsymbol{gxe}_{\boldsymbol{y}}}$ **+**$\boldsymbol{\varepsilon}_{\mathbf{y}}$ Equation S1

**c=** $\mathbf{X}\boldsymbol{\beta}_{\mathbf{add}_{\mathbf{c}}}$ **+** $\boldsymbol{\varepsilon}_{\mathbf{c}}$ Equation S2

where all notations are already defined in the Main text (see Methods).

For the reverse direction, the model can be written as

**c = X**$\boldsymbol{\beta}_{\mathbf{add}_{\mathbf{c}}}$ **+ y**$\beta_{y}$ **+** (**X⊙ y**) $\boldsymbol{\beta}_{\boldsymbol{gxe}_{\boldsymbol{c}}}$ **+**$\boldsymbol{\varepsilon}_{\mathbf{c}}$ Equation S3

where **c** and **y** represent the n× 1 vectors of outcome and exposure, for the reverse direction, respectively. **X** is the n × m genotype matrix, where *m* is the number of single nucleotide polymorphisms (SNPs). $\boldsymbol{\beta}_{\mathbf{add}_{\mathbf{c}}}$ and $\boldsymbol{\beta}_{\boldsymbol{gxe}_{\boldsymbol{c}}}$ represent the additive and non-additive genetic effects of the m SNPs, $\beta_{y}$ is the estimated effect of the exposure on **c**, and $\boldsymbol{\varepsilon}_{\mathbf{c}}$ is the n × 1 vector of residual effects. The Hadamard product (⊙) signifies element-wise multiplication of vectors.

From Equation S3, the GxE interaction effects can be estimated as

${\hat{\boldsymbol{\beta}}}_{\boldsymbol{gxe}_{\boldsymbol{c}}}\mathbf{=}\mathrm{cov}\mathbf{(c,}[\mathbf{X}\odot\mathbf{y])/}\mathrm{var}\mathbf{(X}\odot\mathbf{y}]\mathbf{)}$ Equation S4

Expanding the numerator and substituting **y** from Eq. S1 yields

$\mathrm{cov}\left( \mathbf{c,}\left[ \mathbf{X}\odot\mathbf{y} \right] \right)\boldsymbol{=}\boldsymbol{cov}\left( \mathbf{c}\boldsymbol{,}\left[ \boldsymbol{X}\odot\left( \mathbf{X}\boldsymbol{\beta}_{\boldsymbol{add}_{\boldsymbol{y}}} \mathbf{+ c}\beta_{y}\mathbf{+}\left( \boldsymbol{X\odot c} \right)\boldsymbol{\beta}_{\boldsymbol{gxe}_{\boldsymbol{y}}} \mathbf{+}\boldsymbol{\varepsilon}_{\mathbf{y}} \right) \right] \right)$

Because **c** appears on both sides of this covariance expression, non-zero covariance terms are introduced even when the true $\boldsymbol{\beta}_{\boldsymbol{gxe}_{\boldsymbol{c}}}$**=** 0. This leads to spurious GxE interaction (i.e., var(${\hat{\boldsymbol{\beta}}}_{\boldsymbol{gxe}_{\boldsymbol{c}}}$) > 0) in the reverse direction model, unless $\boldsymbol{\beta}_{\boldsymbol{gxe}_{\boldsymbol{y}}}$**=** 0 in the proposed model. This has been explicitly verified in simulations (see Fig 1-4). The causal mechanisms for the proposed and reverse direction are illustrated in Fig. S1.

**GCIM method to infer the direction of genetic causality.**

To prevent spurious GxE interaction signals, the Genetic Causality Inference Model (GCIM) removes covariance components involving the environmental factor **c**. Specifically, we replace the observed outcome or exposure with their genetically predicted values (polygenic risk scores, PRS). This ensures that the additive genetic effect $\mathbf{X}\boldsymbol{\beta}_{\boldsymbol{add}_{\boldsymbol{y}}}$ does not contain any component of **c** when evaluating the covariance between *c* and the interaction term $\left( \boldsymbol{X\odot y} \right)$. The genetically predicted value of y is denoted as **ĝ_y_ =** $\mathbf{X}\boldsymbol{\beta}_{\boldsymbol{add}_{\boldsymbol{y}}}$, the estimated PRS of the additive genetic effects of **y.**

Accordingly, the reverse-direction GCIM model is written as:

**c = X**$\boldsymbol{\beta}_{\mathbf{add}_{\mathbf{c}}}$ **+ y**$\beta_{y}$ **+** (**X⊙ ĝ_y_**) $\boldsymbol{\beta}_{\boldsymbol{gxe}_{\boldsymbol{c}}}$ **+**$\boldsymbol{\varepsilon}_{\mathbf{c}}$ Equation S5

Similary, the model for the proposed direction can be written as:

**y =** $\mathbf{X}\boldsymbol{\beta}_{\boldsymbol{add}_{\boldsymbol{y}}}$ **+ c**$\beta_{c}$ **+** (**X⊙ ĝ_c_**) $\boldsymbol{\beta}_{\boldsymbol{gxe}_{\boldsymbol{y}}}$ **+**$\boldsymbol{\varepsilon}_{\mathbf{y}}$ Equation S6

Testing both directions using this structure allows us to identify the correct causal direction.

From Equation S5, the GxE interaction effects can be estimated as

${\hat{\boldsymbol{\beta}}}_{\boldsymbol{gxe}_{\boldsymbol{c}}}\mathbf{=}\mathrm{cov}\mathbf{(c,}[\mathbf{X}\odotĝ_{y}\mathbf{])/}\mathrm{var}\mathbf{(X}\odotĝ_{y}]\mathbf{)}$ Equation S7

Because $ĝ_{y}$is derived solely from genotypes and contains no information about c, and c is regressed on y to adjust for shared genetic and environmental as in Eq. 5,

$\mathrm{cov}\mathbf{(c,}[\mathbf{X}\odotĝ_{y}\mathbf{])}$ **= 0**

under the null hypothesis of no G×E interaction in the reverse direction.

This holds because:

1. $ĝ_{c}$​ is a deterministic function of X and independent of c and its residual $\boldsymbol{\varepsilon}_{\mathbf{c}}$;
2. $\mathbf{X}\odotĝ_{y}$ is therefore purely genetic; and
3. after adjusting **c** for **y**, **c** is uncorrelated with any purely genetic term of y.

Consequently, GCIM avoids spurious G×E interaction signals and enables valid inference of the causal direction between genetically correlated traits. By construction, GCIM also minimizes the risk of collider bias, as the PRS are estimated independently of outcome residuals and contain only genetic variation. Substituting PRS for the observed phenotype thus removes shared environmental or causal dependencies between **c** and **y**, ensuring unbiased estimation of G×E effects and valid identification of the true causal direction.

While GCIM provides results that are consistent with causal interpretation, it does not follow the standard causal inference framework that involves specifying untestable assumptions, formally proving identification, and estimating a causal parameter. Instead, GCIM offers an empirical test of directional asymmetry that aligns with a causal interpretation if certain key assumptions hold, notably:

- Exogeneity of the exposure PRS: The exposure PRS is not affected by the outcome or unmeasured confounders of the outcome.
- Exclusion restriction: The exposure PRS affects the outcome only through the exposure, with no direct pleiotropic effects.
- Correct model specification: GCIM adequately models relevant G×E interactions and adjusts for the observed associations between exposure and outcome.

Under these assumptions, the GCIM framework provides a principled and empirically grounded approach to testing the direction of genetic causality.

We have generated GCIM from the current PRSxE approach by substituting the exposures PRS for all exposure values, as demonstrated in Equations S5 and S6 for the general GxE model.


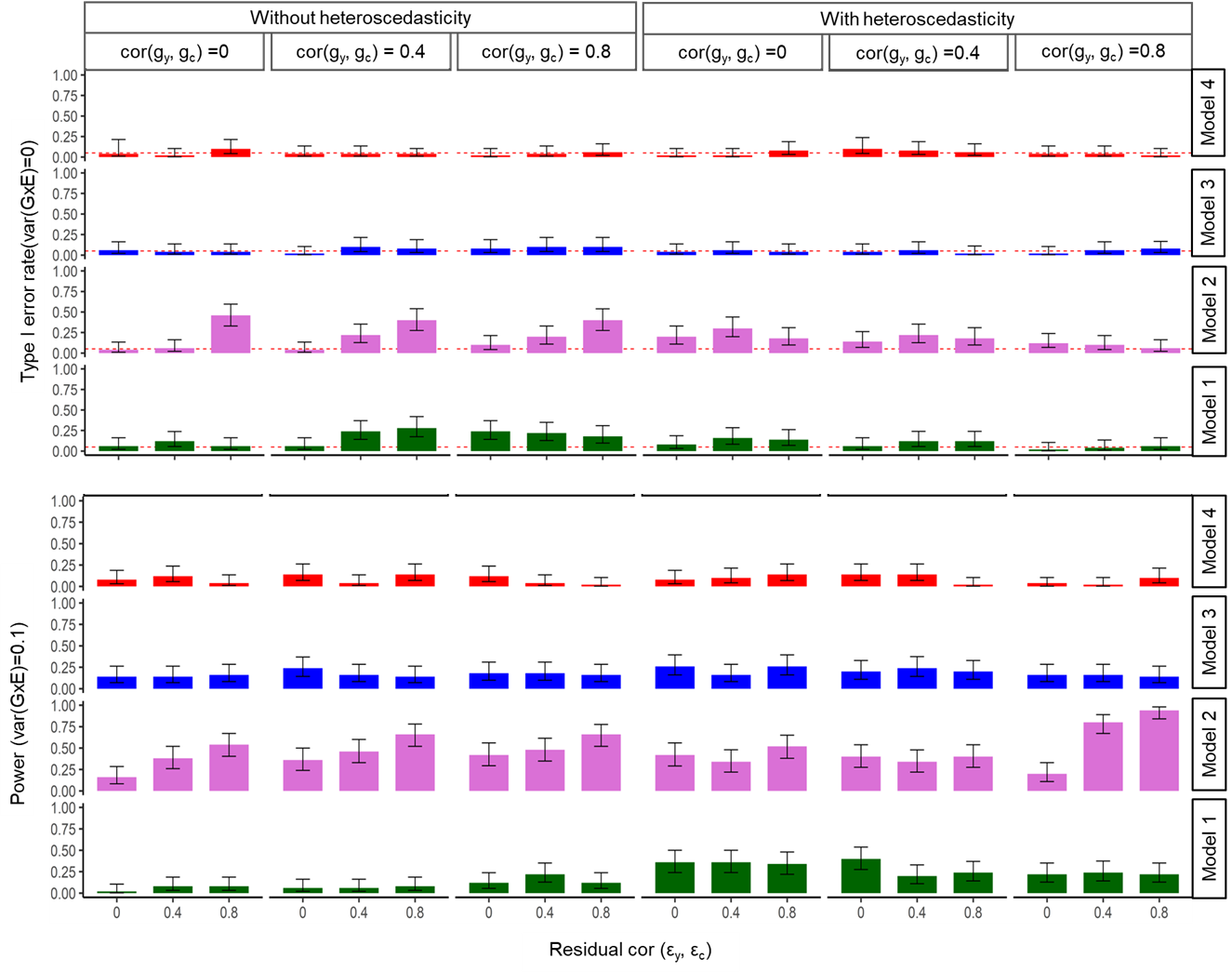


**Fig S2** Simulation results for a binary outcome with a binary exposure under the true causal direction**.** Results are shown across varying genetic correlations and residual correlations, under scenarios with and without heteroscedasticity (i.e. residual-by-environment interactions). The top panel reports type I error rates under the null, and the bottom panel shows power under the alternative. Within each panel, bars are grouped by genetic correlation cor$(\mathbf{g}_{\boldsymbol{y}},\mathbf{g}_{\boldsymbol{c}})$= 0, 0.4, 0.8. For each genetic correlation, the three bars correspond to residual correlations of cor$(\boldsymbol{\varepsilon}_{\mathbf{y}}\mathbf{,}\boldsymbol{\varepsilon}_{\mathbf{c}})$ = 0, 0.4, and 0.8. This structure illustrates how correlation and heteroscedasticity jointly affect method performance. Model 4 represents the proposed model (GCIM), whereas Models 1–3 correspond to existing methods.


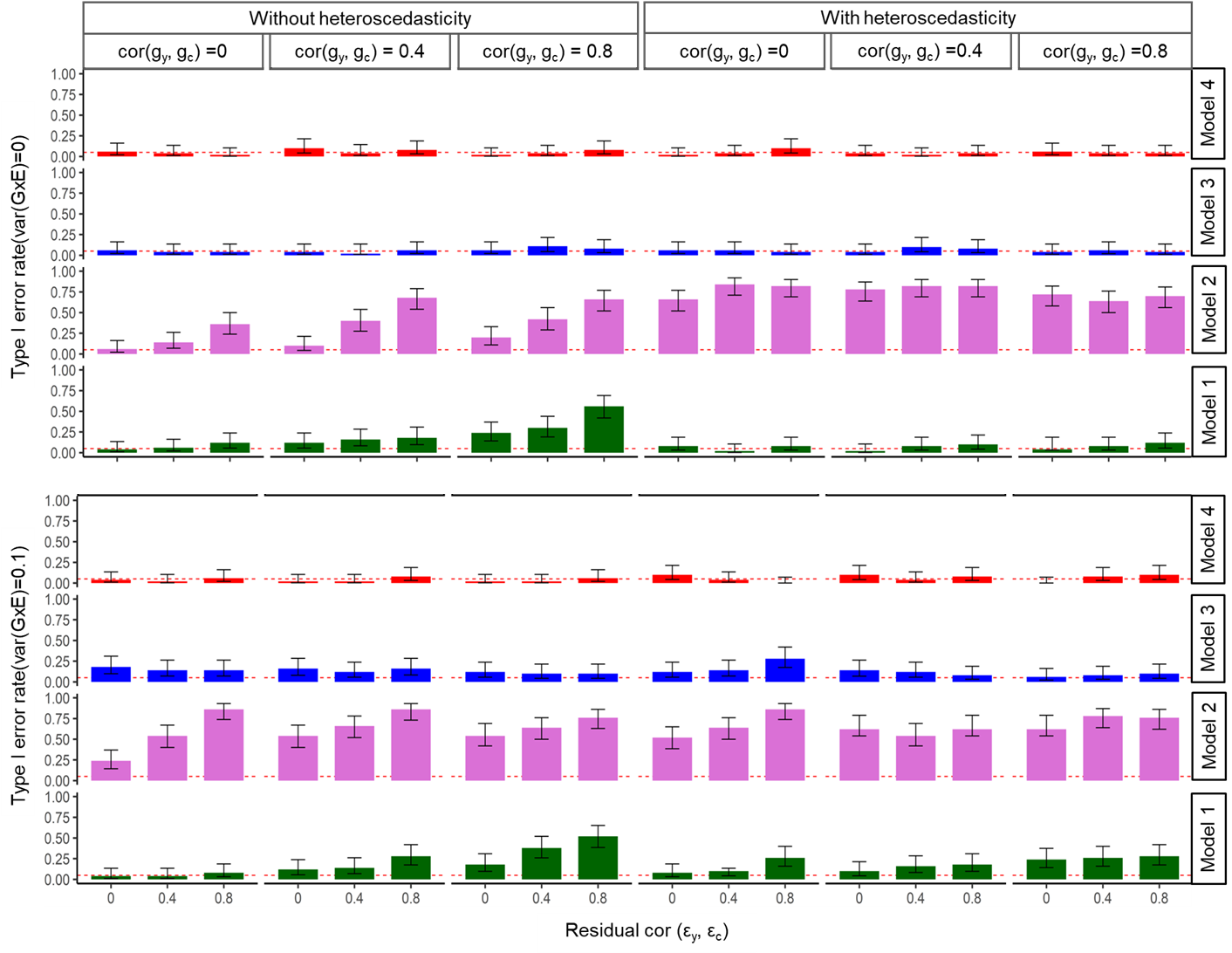


**Fig S3** Simulation results for binary outcome and binary exposure under reverse causal direction. Results are shown across varying genetic correlations and residual correlations, under scenarios with and without heteroscedasticity (i.e. residual-by-environment interactions). The top panel reports type I error rates under the null, and the bottom panel shows power under the alternative. Within each panel, bars are grouped by genetic correlation cor$(\mathbf{g}_{\boldsymbol{y}},\mathbf{g}_{\boldsymbol{c}})$= 0, 0.4, 0.8. For each genetic correlation, the three bars correspond to residual correlations of cor$(\boldsymbol{\varepsilon}_{\mathbf{y}}\mathbf{,}\boldsymbol{\varepsilon}_{\mathbf{c}})$ = 0, 0.4, and 0.8. This structure illustrates how correlation and heteroscedasticity jointly affect method performance. Model 4 represents the proposed model (GCIM), whereas Models 1–3 correspond to existing methods.


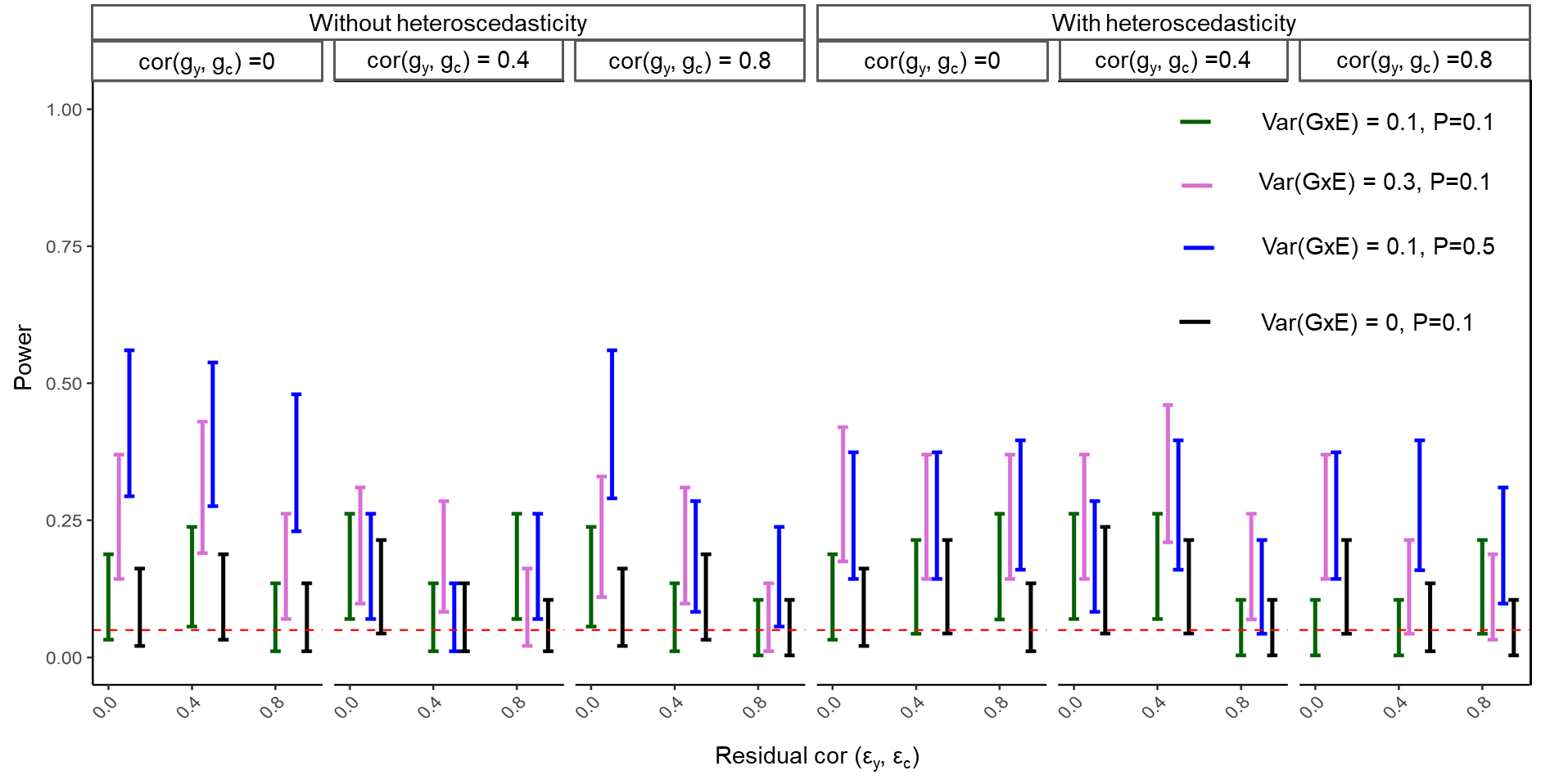


**Fig S4** Simulation results for binary outcome and binary exposure analysed showing statistical power for Model 4 (GCIM). Results are shown across varying genetic correlations and residual correlations, under scenarios with and without heteroscedasticity (i.e. residual-by-environment interactions). The panel shows power under the alternative model: green (GxE variance = 0.1, prevalence = 0.1), pink (GxE variance = 0.3, prevalence = 0.1), blue (GxE variance = 0.1, prevalence = 0.5), compared with black error bars of the null model (prevalence = 0.1). Within the panel, bars are grouped by genetic correlation cor$(\mathbf{g}_{\boldsymbol{y}},\mathbf{g}_{\boldsymbol{c}})$= 0, 0.4, 0.8. For each genetic correlation, the three bars correspond to residual correlations of cor$(\boldsymbol{\varepsilon}_{\mathbf{y}}\mathbf{,}\boldsymbol{\varepsilon}_{\mathbf{c}})$ = 0, 0.4, and 0.8. This structure illustrates how correlation and heteroscedasticity jointly affect method performance.


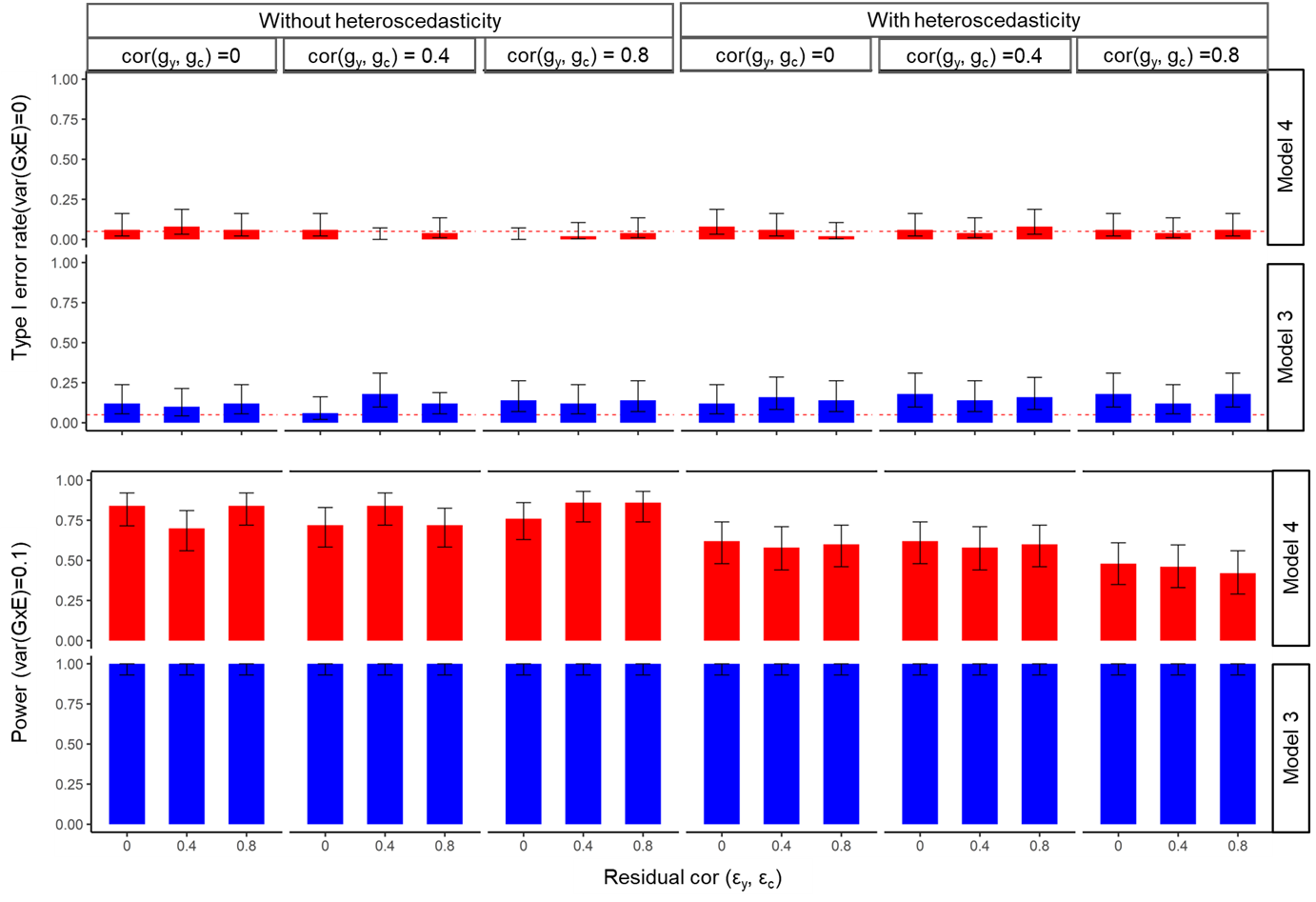


**Fig S5** Simulation results for quantitative outcome and quantitative exposure analysed without the main effect of exposure. Results are shown across varying genetic correlations and residual correlations, under scenarios with and without heteroscedasticity (i.e. residual-by-environment interactions). The top panel reports type I error rates under the null, and the bottom panel shows power under the alternative. Within each panel, bars are grouped by genetic correlation cor$(\mathbf{g}_{\boldsymbol{y}},\mathbf{g}_{\boldsymbol{c}})$= 0, 0.4, 0.8. For each genetic correlation, the three bars correspond to residual correlations of cor$(\boldsymbol{\varepsilon}_{\mathbf{y}}\mathbf{,}\boldsymbol{\varepsilon}_{\mathbf{c}})$ = 0, 0.4, and 0.8. This structure illustrates how correlation and heteroscedasticity jointly affect method performance. Model 4 represents the proposed model (GCIM), whereas Model 3 corresponds to an existing method.


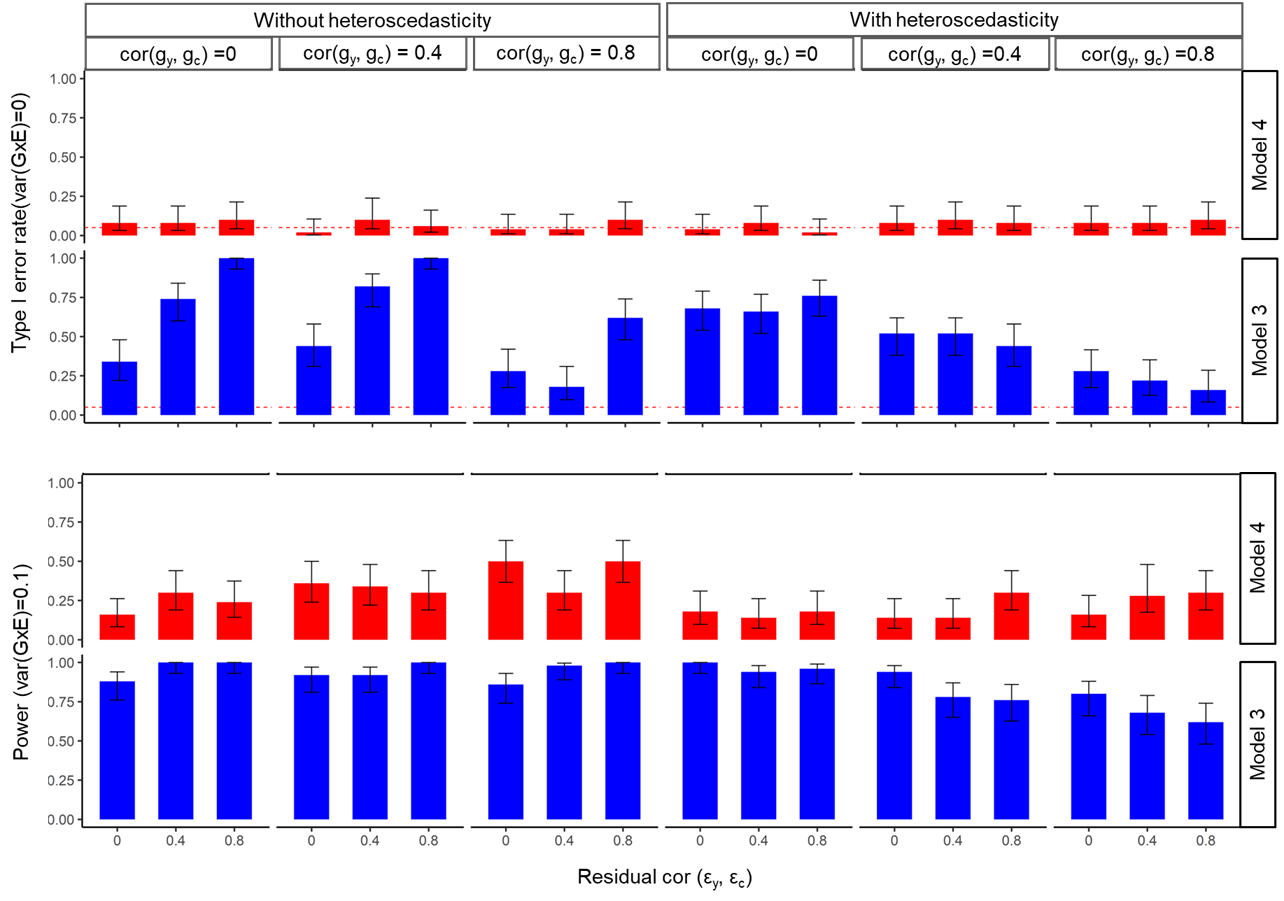


**Fig S6** Simulation results for quantitative outcome and quantitative exposure analysed without the main effect of exposure. Results are shown across varying genetic correlations and residual correlations, under scenarios with and without heteroscedasticity (i.e. residual-by-environment interactions). The top panel reports type I error rates under the null, and the bottom panel shows power under the alternative. Within each panel, bars are grouped by genetic correlation cor$(\mathbf{g}_{\boldsymbol{y}},\mathbf{g}_{\boldsymbol{c}})$= 0, 0.4, 0.8. For each genetic correlation, the three bars correspond to residual correlations of cor$(\boldsymbol{\varepsilon}_{\mathbf{y}}\mathbf{,}\boldsymbol{\varepsilon}_{\mathbf{c}})$ = 0, 0.4, and 0.8. This structure illustrates how correlation and heteroscedasticity jointly affect method performance. Model 4 represents the proposed model (GCIM), whereas Model 3 corresponds to an existing method*.*

References

1. Jayasinghe D, Momin MM, Beckmann K, Hyppönen E, Benyamin B, Lee SH. Mitigating type 1 error inflation and power loss in GxE PRS: Genotype–environment interaction in polygenic risk score models. Genetic Epidemiology. 2024;48(2):85-100.
